# Kinetics thermodynamics and adsorption study of raw treated diatomite as a sustainable adsorbent for crystal violet dye

**DOI:** 10.1038/s41598-025-05787-3

**Published:** 2025-07-01

**Authors:** Mokhtar Saidi, Bendoukha Abdelkrim Reguig, Mohammed El Amine Monir, Talal M. Althagafi, M. Fatmi, Abderahmane Remil, Abdelhafid Zehhaf, M. A. Ghebouli

**Affiliations:** 1 Laboratory of Organic, Macromolecular Chemistry and Materials, Department of Chemistry, Faculty of Exact Sciences, Mascara University, BP 763, Mascara, 29000 Algeria; 2 Faculty of the Exact Sciences, Mustapha Stambouli University of Mascara, B.P. 305, Mascara, 29000 Algeria; 3 Department of Physics, College of Science, Taif University, P.O. Box 11099, Taif, 21944 Saudi Arabia; 4 Research Unit on Emerging Materials (RUEM), University Ferhat Abbas of Setif 1, Setif, 19000 Algeria; 5 Laboratory of Process Engineering and Chemistry Solution, Department of Process Engineering, Faculty of Science and Technologies, Mascara University, Mascara, 29000 Algeria; 6 Department of Chemistry, Faculty of Sciences, University of M’sila University Pole, Road Bourdj Bou Arreiridj, 28000 M’sila, Algeria

**Keywords:** Adsorption, Diatomite, Crystal violet, Acid treatment, Water treatment, Dye removal, Materials science, Optics and photonics

## Abstract

Adsorption is a simple and effective method to remove heavy metal ions and anionic or cationic dyes from aqueous or waste solutions. Herein, we report on the adsorption of a cationic dye crystal violet on a low-cost natural material. The diatomite was obtained from Sig deposit (west of Algeria) and then treated with 1 M HCl solution. Natural clay (diatomite) was characterized using different physical-chemical methods, including, X-ray powder diffraction, X-ray fluorescence, thermogravimetric analysis, scanning electron microscopy, and Fourier transform infrared and pH of zero charge. The effect of various experimental parameters, such as initial dye concentration, adsorbent dose, initial pH, and shaking time on adsorption was investigated. The maximum adsorption capacity was found to be 82.0 mg.g^− 1^ for treated Diatomite and 75.0 mg.g^− 1^ for raw diatomite, with equilibrium reached within 120 min. Adsorption kinetics followed the pseudo-second-order model, with high correlation coefficients (R² = 0.998 for treated diatomite and R² = 0.999 for raw diatomite). The isotherm data showed the best fit with the Langmuir model. Optimal adsorption occurred at pH 8 for treated diatomite and pH 10 for raw diatomite.

**Supplementary Information:**

The online version contains supplementary material available at 10.1038/s41598-025-05787-3.

## Introduction

Water pollution, particularly from industrial effluents, poses a severe threat to the environment and public health^1^. Industries have a major role in effluent discharge that contributes to environmental degradation^2^. Dye, a pollutant and the most visible indicator of water pollution reduces the efficiency of photosynthesis in aquatic plants, affecting plant growth^3^. In addition, they are carcinogenic and mutagenic to aquatic life as well as human health, being toxic even at very low concentrations in water^4^. Dyes such as Crystal Violet (CV), a cationic triphenylmethane dye extensively used in industries like textiles, plastics, cosmetics, paper, and pharmaceuticals, are particularly concerning due to its highly genotoxic, toxic, mutagenic and carcinogenic nature^5^. During the manufacturing process, the textile sector rejects a lot of colored effluent into the wastewater, which amounts to about 40% of the total dye used^6^.

With the growing global shortage of clean drinking water, the water crisis has become a critical concern^7^. In recent years, various advanced water treatment methods have been developed to mitigate the water crisis, including advanced oxidation processes^8^, biodegradation^9^, Photo-catalytic^10^ and desalination technologies^11^. However, some of these methods have downsides such as high treatment costs, technical limitations, and limited utilization^12^. Therefore, scientists are increasingly interested in developing less expensive and more efficient methods^13^. As a result, methods like adsorption might be viewed as an intriguing alternate option for dealing with this sort of pollutant. The adsorption process is one of the most significant methods to remove dyes from wastewater in terms of environmental compatibility, very effective, simple, low operating cost, and high efficiency^12,14^.

A variety of adsorbents can be used for this purpose^15^. Activated carbon is a typical adsorbent for dyes removal from aqueous solution because it has a high adsorption capacity due to its porous structure and large surface area^16^. However, the expensive cost and difficulties of regeneration, activated carbons are not suitable for large-scale use^17^. Therefore, a significant number of research has been conducted in recent years to find low-cost, efficient, and widely available adsorbents such as mesoporous materials^18^, biomass^19^, nanocomposite^20^, clays^21^, zeolites^22^ and other adsorbents^23^.

Diatomite is a siliceous rock formed by fossil accumulation of diatom shells^24^. The structure is amorphous containing mainly SiO_2_.nH_2_O^25^. Diatomite has unique physical and chemical properties (surface area, porosity, and excellent thermal and mechanical stability) which make it used in various fields of sustainable development and environment such as purification of drinking water, manufacture of antibiotics, additive in cement, filtering medium for a number of industrial applications and the removal of inorganic and organic pollutants from wastewater due to its abundance, low cost and availability^26–28^. To date, diatomite has been utilized in adsorption studies for the removal of heavy metals Pb(II) and Cd(II)^29,30^ and dyes CV 2B dye^31^, methylene blue^32^ However, to increase the adsorption capacity of dyes, treatments with various methods such as chemical and physical modifications, calcinations, inorganic bases, surfactants, and salts have been used to modify the surface and pore structure of diatomite^33^. Among these methods, hydrochloric acid (HCl) treatment is particularly effective, as it increases the surface area and porosity by removing impurities and enhances the surface acidity, improving electrostatic interactions between the adsorbent and cationic dye molecules, thereby boosting the overall adsorption efficiency^34^.

In this study, we propose the utilization of a low-cost natural material (diatomite), which is widely available in Sig deposit (west of Algeria), and the application of inorganic acid treatment as an efficient method to modify the surface of diatomite and to increase the removal efficiency of CV from aqueous solution. CV is widely employed in the Algerian textile industry, as well as in cosmetics, plastics, photographic, and also paper industries. Surface characteristics of the diatomite and its modified counterpart were evaluated using XRD, SEM, XRF, FTIR, GTA, pH_PZC_ and BET analyses. The significance of this work lies in the application of an efficient and eco-friendly adsorbent based on locally sourced diatomite enhanced by acid treatment, resulting in improved surface properties and a significantly higher adsorption capacity compared to materials reported in the literature. The main objectives of this study are to characterize the raw and treated diatomite, evaluate the influence of key operational parameters on CV dye adsorption, and model the adsorption process through kinetic, isotherm, and thermodynamic analyses.

## Materials and methods

### Chemicals and materials

The Diatomite used in this work is collected from Sig deposit in Mascara (West of Algeria), NaOH (98%), HCl (37%), and AgNO_3_ (99.8%) were purchased from Sigma-Aldrich (Munich, Germany).

### Adsorbent

**Fig. 1:**
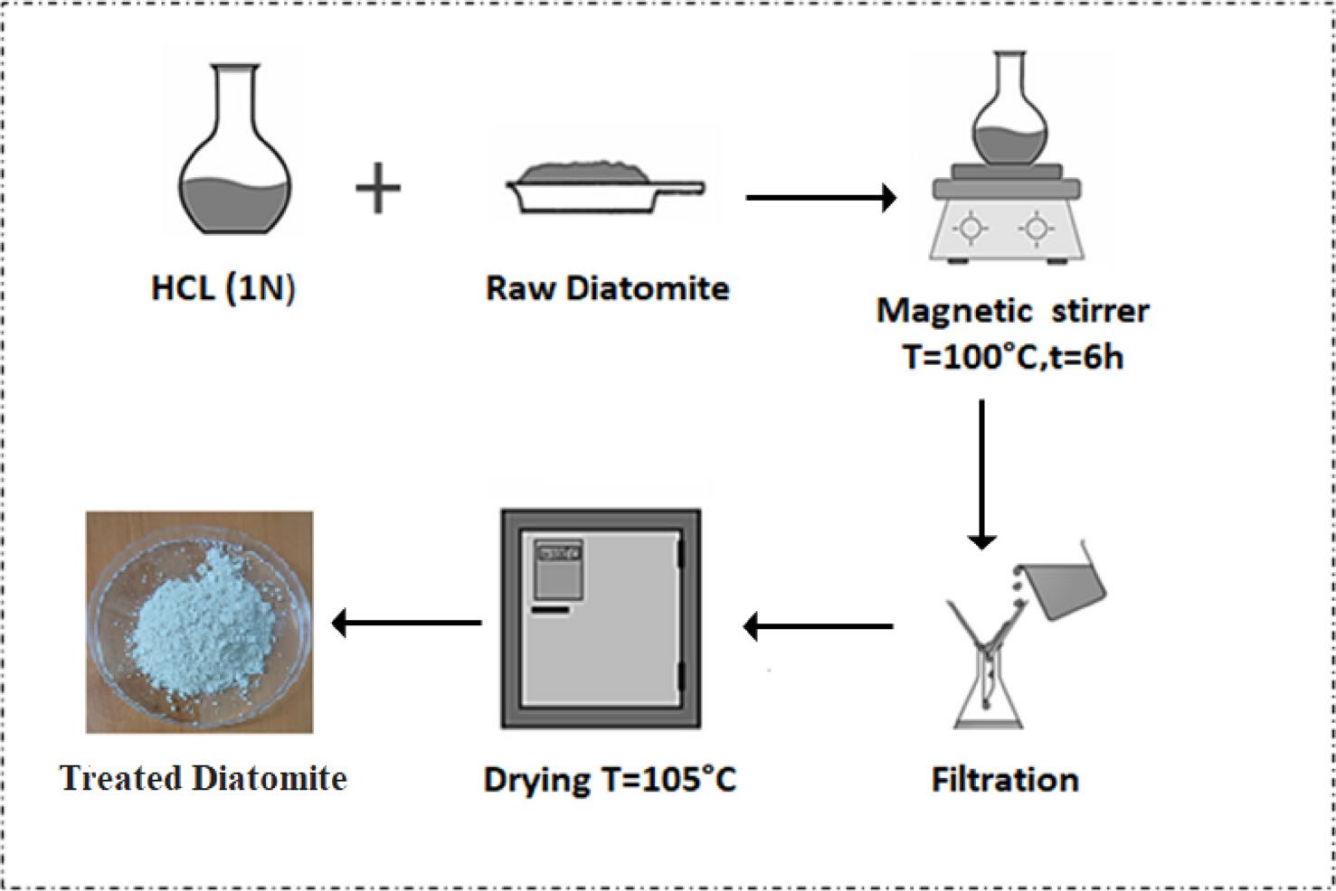
Method of preparation of treated diatomite DT.

Raw Diatomite denoted as RD used in this work is collected from Sig deposit in Mascara (West of Algeria). The sample amount of 5 g was added to 100 mL of HCl solution at normality 1 N, and refluxed at 100 °C for six hours’ time contact. Distilled water was used to wash the sample to remove the HCl. Titration with 0.1 M silver nitrate (AgNO_3_) was conducted on the wash water to check for residual chloride ions, indicated by the formation of a white precipitate of silver chloride (AgCl). Washing continued until no precipitate was formed, confirming the removal of chloride ions. The treated diatomite was then dried at 105 °C and referred to as DT (treated diatomite), presented in Fig. 1.

### Adsorbate

The chemical formula of CV is C_25_H_30_N_3_Cl, and its molecular weight is 407.99 g/mol.

The IUPAC name of CV is 4-{Bis [4-(dimethylamino)phenyl]methylidene}-N, N-dimethylcyclohexa-2,5-dien-1-iminium chloride and its CAS number is 548-62-9. The solubility of CV in water is16 g. L^− 1^ at 25 °C. were purchased from Sigma Aldrich.

### Characterization methods

The SEM was used to visualize the surface morphology of diatomite and its modified counterpart, using a « FEI NovaNanoSEM 230 microscope » operating at 8 kV. The chemical compositions of diatomite were determined by (XRF) (S8 TIGER de Bruker). The thermal (TGA) properties of obtained materials were performed by MOM Derivatograph Q1500Dunder air atmosphere in the temperature range (25–800) °C with a heating rate of 5 °C.min^− 1^. (XRD) data were obtained using a BrukerD4 ENDEAVOR analyzer with a CuKa radiation source filtered with a graphic monochromator *λ* = 1.5406.(FTIR) was recorded on a Perkin-Elmer infrared spectrophotometer in the range of 400–4000 cm^− 1^. The specific surface area (S_BET_) of diatomite before and after activation was determined by the BET method using N2 adsorption (Micromeritics Gemini VII 1014 Instrument).

### Adsorption experiments

The efficiency of the removal of CV on diatomite clay from aqueous solution was studied through batch methods. Firstly, 1 g of CV was dissolved in distilled water to prepare the aqueous stock solution at a concentration of 1000 mg.L^− 1^. After that, it was diluted to various desired concentrations, and the pH of the solutions was then adjusted using HCl and NaOH (0.1 M). The effect of various parameters like initial pH (3–10), adsorbent mass (10–70 mg), dyes concentration (20–120 mg.L^− 1^), and the temperature effect (298–313) K on the CV sorption rate were examined. Each Adsorption experiment was carried out in a series of 100-ml Erlenmeyer flasks, to which 50 mg of diatomite was mixed with 50 mL of a specified initial concentration dye solution. The suspensions were shaken in a GFL Type 1083 shaker for 120 min to reach equilibrium at room temperature (298 K). After completing the adsorption process, the mixture was centrifuged at 4000 rpm for 5 min to obtain the supernatant liquid. After adsorption, the residual amounts of CV in the solution were determined using a UV-VIS spectrophotometer (VIS 7220G) at the λmax = 584 nm. All these experiments were repeated three times for each parameter investigated to ensure accuracy and reliability of the results. The adsorption capacity (*q*_*e*_) and the removal efficiency (R %) of CV in the removal process were calculated by using Eqs. (1) and (2) in the Sect. 2.6, respectively.

### Theoretical calculations and models

The adsorption mechanism was analyzed using the following theoretical frameworks:

## Adsorption capacity & efficiency

1 $$\:{q}_{e}=\frac{{C}_{0}-{C}_{e}}{\text{m}}\times\:v$$

2 $$\:R\%=\frac{{C}_{0}-{C}_{e}}{{C}_{0}}\times\:100$$

Where C_0_ is the dye’s initial concentration (mg.L^− 1^), C_e_ is the dye’s equilibrium concentration.

(mg. L^− 1^), V is the CV solution’s volume (L), and m is the adsorbent mass used in this experiment (g).

## Kinetic models

The nonlinear forms of pseudo-first-order, pseudo-second-order, Elovich and intraparticle diffusion models is given in Eqs. (3), (4), (5) and (6) respectively:

3 $$~q_{t} = q_{e} (1 - e^{{k_{1} t}} )~$$

4 $$\:\:{q}_{t}=\frac{{k}_{2}{{q}_{e}}^{2}t}{(1+{k}_{2}{q}_{e}t)}\:\:\:\:\:\:\:\:\:\:\:\:\:\:\:\:\:\:\:\:\:\:\:\:\:\:\:\:\:\:\:\:\:\:\:\:\:\:\:\:\:\:\:\:$$

5 $$\:{\text{q}}_{\text{t}}=\frac{1}{\beta\:}\text{ln}\left(\alpha\:\beta\:t+1\right)\:\:\:\:\:\:\:\:\:\:\:\:\:\:\:\:\:\:\:\:\:\:\:\:\:\:\:\:$$.

Where α and β are coefficients of the Elovich kinetic model and are initial adsorption rate (g/mg.min) and the desorption constant (mg/g), respectively.

6 $$\:{q}_{t}={K}_{id}{t}^{0.5}+C\:$$.

Where K_id_ and C are the intraparticle diffusion rate constant (mg/g. min^0.5^) and boundary layer thickness respectively.

## Isotherm models

The nonlinear forms of the Langmuir, Freundlich, and D–R equations are given in Eqs. (7), (8) and (9), respectively:

7 $$\:{q}_{e}=\frac{{q}_{max}{+K}_{L}{C}_{e}}{{1+K}_{L}{C}_{e}}$$

8 $$\:{q}_{e}={K}_{\text{F}}{\left({C}_{e}\right)}^{1/n}$$

9 $$\:{q}_{e}\:=\:{q}_{m}{e}^{-\:\beta\:{\epsilon\:}^{2}}$$

The dimensionless separation factor (RL) for the Langmuir model is calculated by:

10 $$\:RL=\frac{1}{1+{{\text{K}}_{\text{L}}\text{C}}_{0}}$$

The mean free energy of adsorption (E) for the D–R model is given by:

11 $$\:E=\frac{1}{\sqrt{2\beta\:}}$$

where q_max_ (mg.g^− 1^) is the maximum adsorption capacity, K_L_ (L/g) is the Langmuir’s constant that is related to the active sites and adsorption energy, K_F_ (mg/g) and n are constants of Freundlich model, the K_F_ denotes the adsorption capacity and n is the adsorption intensity, β (mol^2^/J^2^) is an activity coefficient indicating free adsorption energy, and ε ( $\:{\upepsilon\:}=RT\:ln\:(1+\frac{1}{Ce}\:)$) is the Polanyi potential.

## Thermodynamic analysis

Gibbs free energy change ($\:\varDelta\:G^\circ\:$), enthalpy ($\:\varDelta\:H^\circ\:$), and entropy ($\:\varDelta\:S^\circ\:$) were determined via:

12 $$\:\varDelta\:G^\circ\:=\varDelta\:H^\circ\:-T\varDelta\:S^\circ\:$$

13 $$\Delta G^\circ = - RT\ln Kd$$

By inserting Eq. (12) and Eq. (13)

14 $$\:lnKd=\frac{\varDelta\:S^\circ\:}{R}-\frac{\varDelta\:H^\circ\:}{RT}\:\:\:\:\:\:\:\:\:\:\:\left(14\right)$$

Where R is the universal gas constant at (8.314 J mol ^− 1^ K^− 1^), T is the absolute temperature on Kelvin, and Kd denotes the equilibrium constant determined using Eq. (15).

15 $$\:Kd=\left(\frac{{C}_{i\:\:\:\:}-{C}_{e}}{{C}_{e}}\right)\:\:\:\:\:\:\:\:\:\:\:\:\:\:\left(15\right)$$

## Results and discussion

### Characterization of the sorbents

The Characterization of diatomite materials before and after modification with HCl (1 M) is crucial in ensuring optimal adsorption capacity. To ensure a cost-effective absorbable material, it is imperative to carefully select the adsorbent material^35,36^. An in-depth understanding of the adsorption process can be achieved by thoroughly characterizing the material being studied. This characterization should include evaluations of the material’s structure, texture, and chemical composition. As a result, a comprehensive analysis of multiple parameters was conducted on the DT and RD materials being scrutinized.

**Fig. 2:**
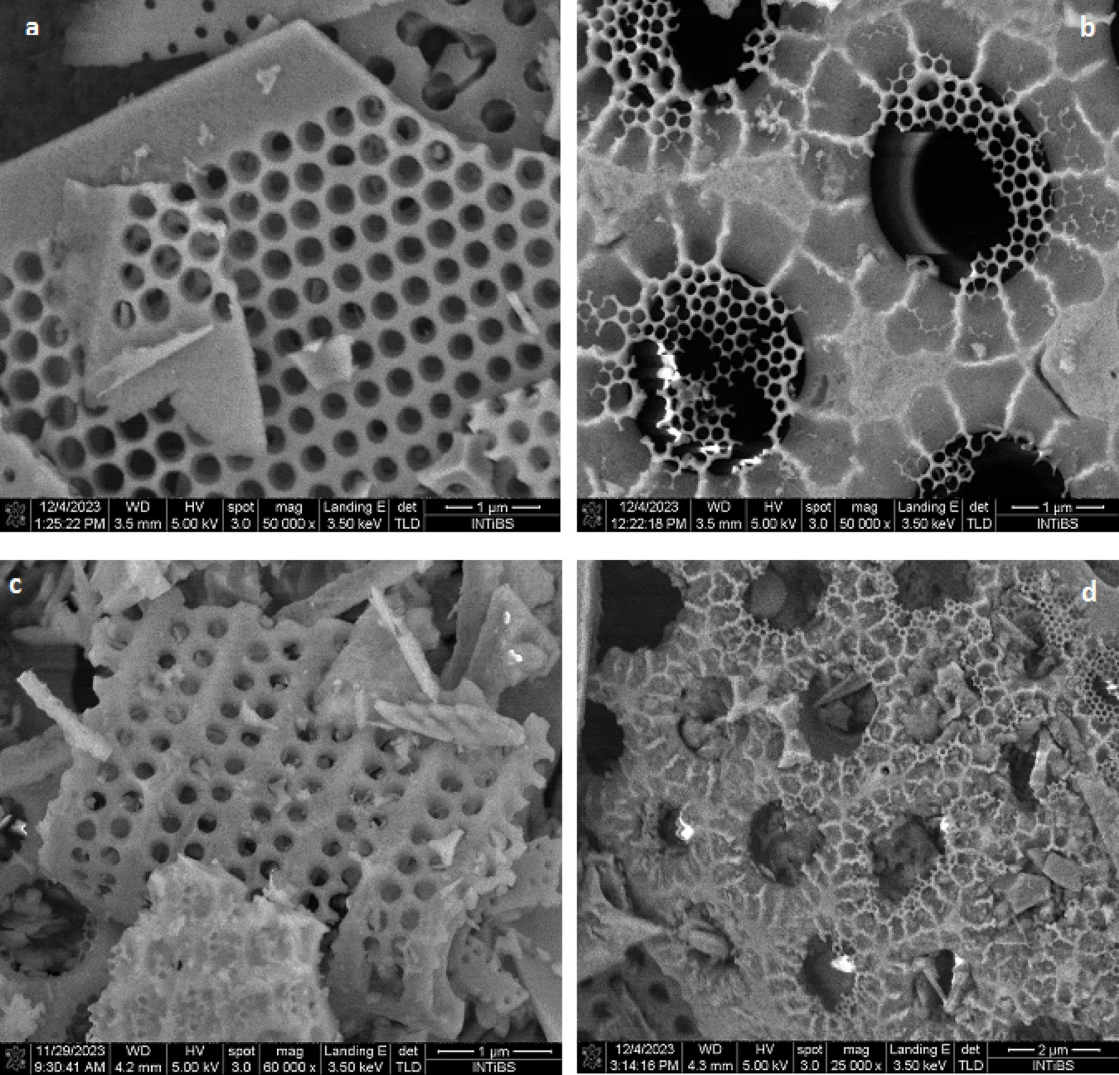
SEM images of diatomite (**a**) treated diatomite before adsorption, (**b**) treated diatomite after adsorption (**c**) raw diatomite before adsorption, (**d**) raw diatomite after adsorption).

The SEM is effectively employed to analyze the physical properties of the adsorbent’s surface, as demonstrated in Fig. 2. SEM images (Fig. 2a) and (Fig. 2c) reveal porous structures in both raw and treated diatomite samples, exhibiting circular shapes akin to honeycombs^37^. Also, an evident comparison of images (Fig. 2b) and (Fig. 2d) highlights CV particle coverage on parts of the untreated diatomite surface, whereas the majority of the treated surface is occupied by the dye (CV). Despite a higher total pore volume; the untreated diatomite exhibits a lower specific surface area than the treated counterpart. This distinctive characteristic acquired by the treated diatomite allows it to provide channels capable of retaining multiple layers of dyes, enhancing the deposition of CV particles on its surface. Overall, SEM analysis proves insightful in understanding the interaction of diatomite with CV.

**Fig. 3:**
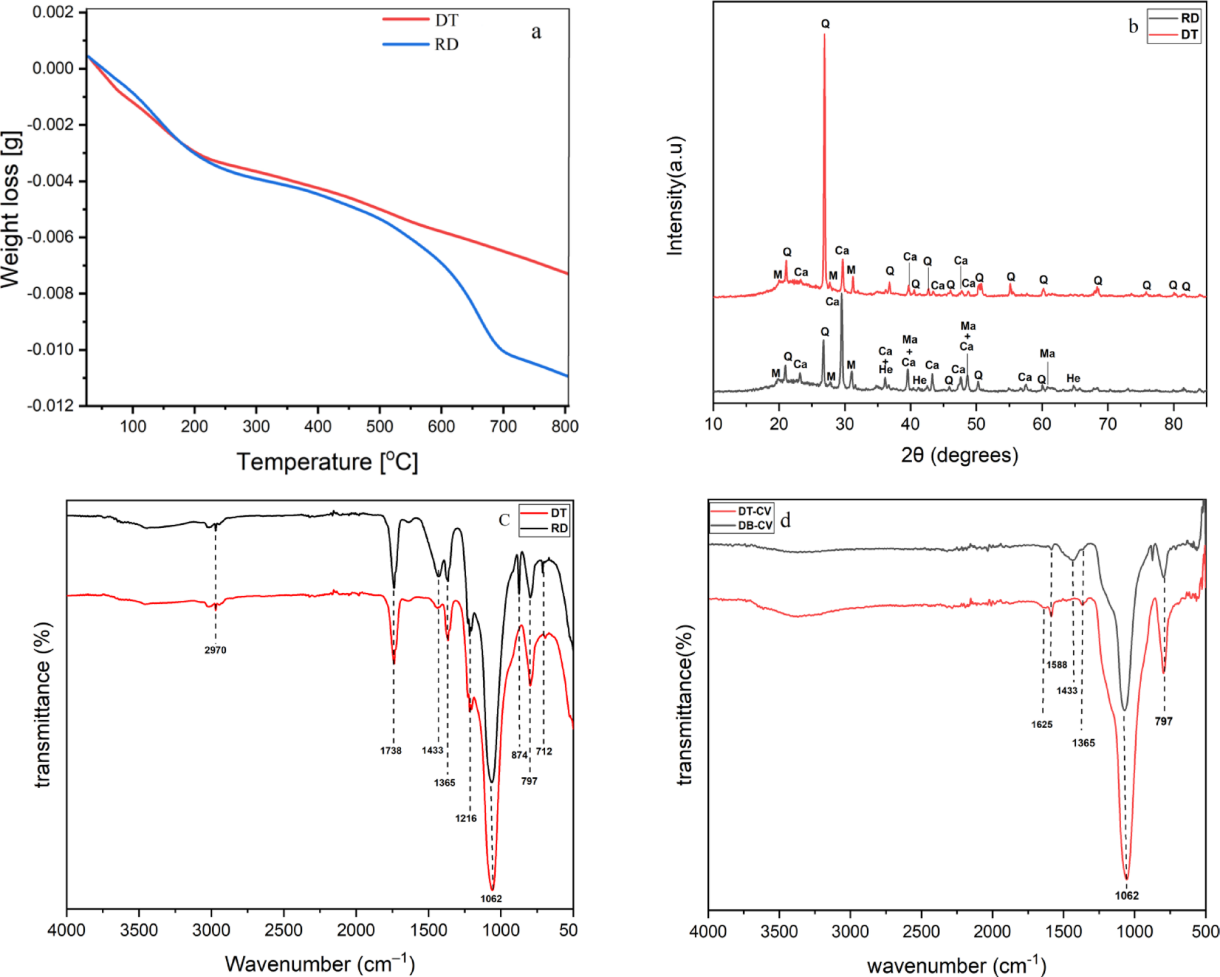
TGA analysis curves of raw and treated diatomite (**a**); (**b**) X-ray patterns of raw and treated diatomite (Q: quartz (SiO_2_), Ca: calcite (CaCO_3_), Mu: muscovite (K (Al_3_Si_3_O_10_) (OH) _2_), He: hematite (Fe_2_O_3_), Ma: magnetite (Fe_3_O_4_); (**b**) FTIR spectra of raw and treated diatomite before CV adsorption; (d) FTIR spectra of raw and treated diatomite after CV adsorption.

Figure 3a; shows the TGA curves of raw and treated diatomite at heating rate 5 °C/min from room temperature to 800 °C, using 70.1 mg of each sample for the analysis. Exhibits three main mass losses. An initial minor weight loss is observed below 300 °C, attributed to the desorption of physiosorbed water present in the diatomite^38^. The second mass loss in the range of 300 °C to 500 °C should be attributed to the dihydroxylation of calcium hydroxide^39^. The third significant weight loss occurs between 500 and 700 °C, linked to carbonate decomposition, which is minimal or absent in the treated diatomite, confirming effective carbonate removal^40^. Above 700 °C, the treated diatomite demonstrates enhanced thermal stability, reflecting higher purity and improved thermal properties. These findings suggest that HCl treatment effectively enhances the purity of diatomite, making it more suitable for various industrial applications.

The XRD patterns for DT and RD samples are displayed in Fig. 3b. The diffractogram indicates that the raw diatomaceous earth is primarily composed of an amorphous silica phase that is visible in the 2θ range of 18° to 25°. Some minerals such as muscovite (Mu), magnetite (Ma), and hematite (He) were also identified in the diatomite samples^41^. In addition, two other crystalline phases were detected: (i) SiO_2_ in the quartz (Q) form, with characteristic peaks at 2θ = 21°, 27°, 36°, 46°, 50°, and 55°^42^, and (ii) carbonate in the form of calcite (Ca), with peaks at 2θ = 23°, 29°, 39°, 43°, and 47°^43^. It is noteworthy that the main quartz peak intensities in the XRD pattern noticeably increased and the calcite peaks decreased, while some peaks disappeared while some others with lower intensity appeared after acidic treatment. Furthermore, some of the amorphous silica phases can be transformed into a crystalline structure, indicating that the amount of silica has been increased due to the elimination of some impurities^44^. The results of the study have shown that the acidic treatment did not have a significant impact on the structure of the diatomaceous earth, which is in accordance with the findings reported in previous studies^45^.

FTIR is a very useful analytic technique for studying the structure of the raw and treated diatomite. The FTIR analysis before and after dye adsorption are shown in Fig. 3c, and Fig. 3d, respectively. Before the adsorption of CV, the FTIR spectra exhibited a broad weak band centered at around 2970 cm^− 1^ could be assigned to O-H stretching of either Si-O-H group^46^. The bands at 1062, 797, and 1216 cm^− 1^ were particularly remarked. The 1062 cm^− 1^ band represents the (–Si–O–Si–) elongation of the siloxane group and the band at about 797 cm^− 1^ corresponds to the Al-O-Si vibration. The peak at 1216 cm^− 1^ is attributed to the Si-O bond in Q_3_ sites of diatomite^46^. Furthermore, characteristic carbonate bands (in the form of calcite) were detected at 712, 874, 1365, 1433, and 1738 cm^− 1^. Upon treatment with acid, the peaks at 712, 874, and 1433 cm^− 1^ disappeared, while those at 1365 cm^− 1^ and 1738 cm^− 1^ persisted, possibly due to the organic compounds of carbonate ion (CO_3_^2−^) group vibrations^46,47^. These findings confirm that acid treatment is particularly effective in removing organic impurities, consistent with the results reported in previous studies^43^. The FTIR spectrum further confirmed the CV adsorption mechanism. After the adsorption of CV, some peaks appeared, some disappeared, and others showed a decrease in intensity or a shift in position. New peaks emerged in the region of 1625–1588 cm⁻¹, corresponding to the C = C stretching vibration of the benzene ring, confirming the adsorption of the dye onto the diatomite surface^48^. The O-H stretching vibrations around 2970 cm⁻¹, related to silanol groups, disappeared, indicating an interaction with CV^49^. Additionally, the carbonate bands at 712 and 1738 cm⁻¹, associated with calcite in both raw and treated diatomite, also disappeared. The bands at 874, 1365, and 1433 cm⁻¹ diminished, suggesting that some carbonate groups were removed during adsorption^50^.

The quantification of chemical compositions of the raw and acid-treated diatomite will help us to precise the variation of diatomite structure before and after the chemical treatment. Table 1 presents the weight percentages of the chemical components of natural and acid-treated diatomite samples. The XRF analysis indicates that Si and Ca oxides are the main components of the raw diatomite, with small amounts of Al_2_O_3_, MgO, K_2_O, Fe_2_O_3_, and other oxides and impurities^51^. However, the SiO_2_ ratio increases from 68.5 to 88.8 wt % after acid treatment while the Ca, Al, and Fe oxide contents decrease with the desperation of MgO, K_2_O, TeO_2_, and NiO. This increase in SiO_2_ ratio is due to the relative resistance of silica to acid attack, whereas Mg, K, and Ni oxides are soluble under acidic conditions^52^. Calcium mainly exists in the form of carbonate, which easily decomposes in acidic media, causing a significant decrease in the CaO content from 15.8 to 2.03 wt %. The XRF analysis also confirms that the addition of 1 M HCl is sufficient to remove calcite almost completely, as previously observed by XRD and FTIR.

The removal of carbonates is likely to alter the density and surface properties, specifically the specific surface area and porosity, as discussed in the subsequent paragraph. To illustrate the effect of acid activation, BET analysis of raw and activated diatomite was carried out. The textural parameters of the raw and treated diatomite samples, such as the specific surface area (S_BET_) and total pore volume, were analyzed. The BET analysis shows an improvement in the specific surface area of diatomite after acid activation, increasing from 29.08 to 82.82 m².g^− 1^, along with an increase in total pore volume from 0.145 to 0.343 cm³/g. This augmentation indicates that the hydrochloric acid treatment might increase the interlamellar space of the diatomite surface, implying that more adsorption sites may be accessible to the dye molecules^53^.

**Table 1:** Chemical composition of Raw and treated diatomite sample obtained by XRF analysis.

Elements (%)	Raw diatomite	treated diatomite
SiO_2_	68.5	88.8
CaO	15.8	2.03
Al_2_O_3_	5.68	4.45
Fe_2_O_3_	2.37	0.82
MgO	1.72	---
K_2_O	1.22	---
TeO_2_	1.18	---
NiO	0.69	---
TiO_2_	0.29	0.62
*Other	2.55	3.28

* Other inorganic oxides, which included ZnO, CuO.

### Operating parameters

#### Effect of initial dye pH

**Fig. 4:**
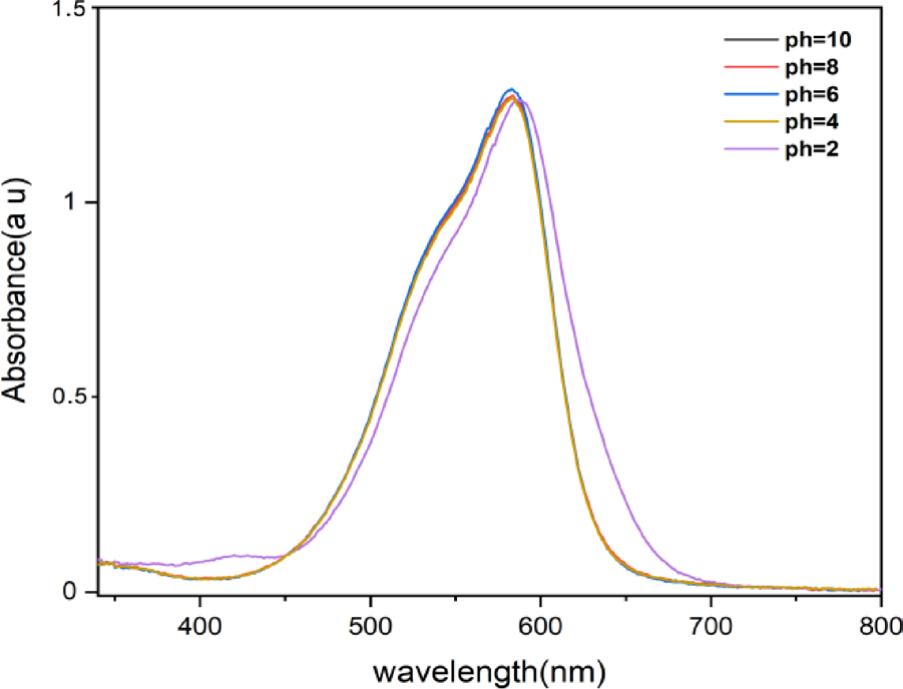
Absorption spectra of CV dye (10 mg L^− 1^) at different pH values.

**Fig. 5:**
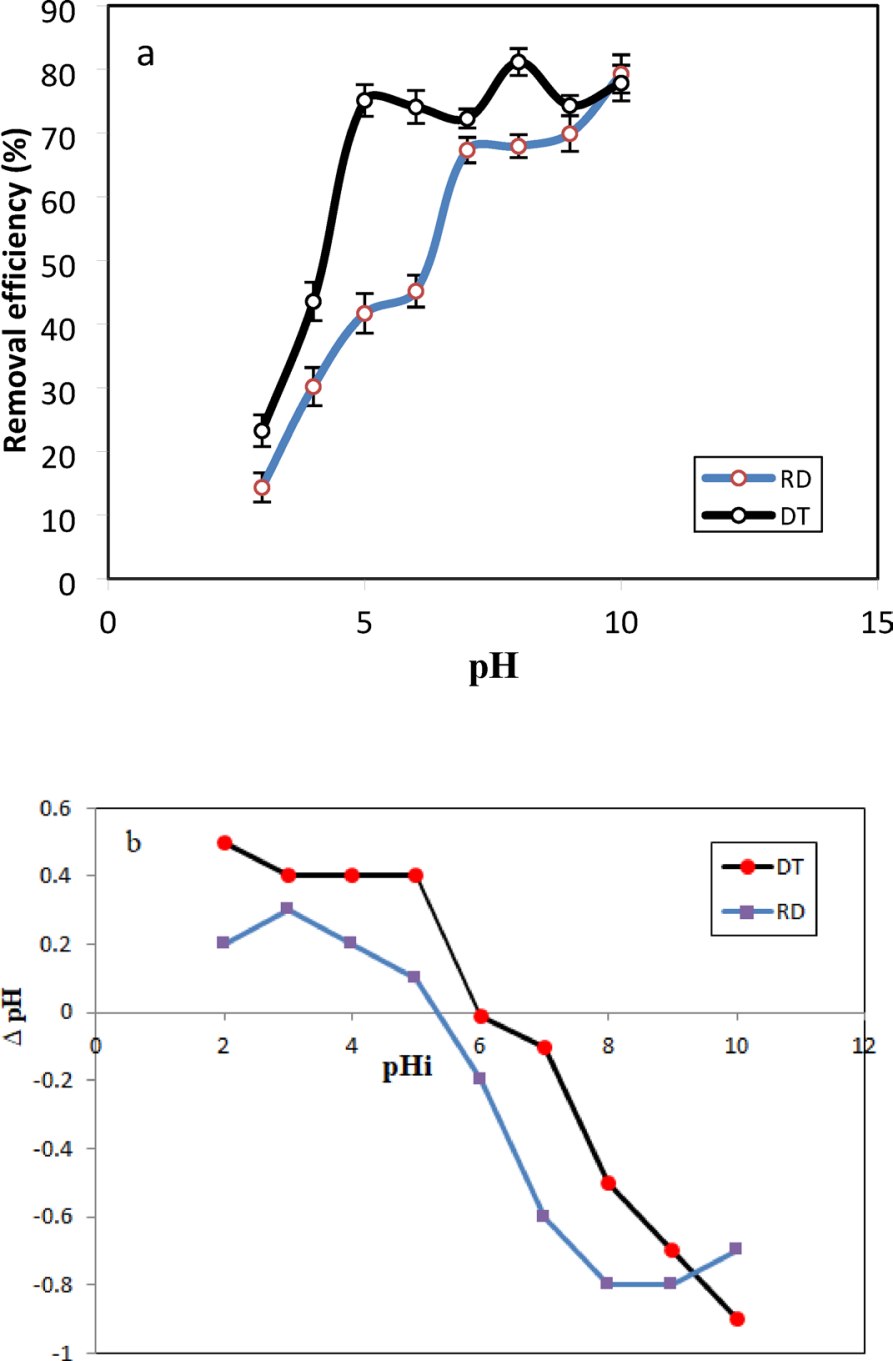
Effect of pH on the removal efficiency of CV on raw and treated diatomite (**a**). Experimental conditions: C_0_ = 40 mg. L^− 1^, m = 40 mg, t = 120 min, T = 25 °C. (** b**) Determination of pH_pzc_ values for raw and treated diatomite.

The pH level plays a significant role in influencing the wavelength of the CV dye^54^. According to Fig. 4, except for a value of pH = 2, the maximum wavelength remains constant across various pH levels. However, a noticeable alteration in wavelength is observed specifically at pH = 2. The impact of the starting pH of dye solution on the adsorption capacity of dye is explored by altering the beginning pH of the dye solution from 3 to 10 under constant process conditions on both materials (RD, DT). Figure 5(a) demonstrates that optimal crystal violet (CV) dye adsorption was achieved under alkaline conditions for both materials examined. The adsorption capacity exhibited a consistent upward trend with increasing pH values specifically from pH 3 to 10 for RD and from pH 3 to 8 for DT, with DT showing a subsequent decline between pH 8 and 10. Maximum sorption efficiency was observed at pH 10 for RD (79.27%) and approximately pH 8 for DT (81.16%). This phenomenon can be attributed to the cationic nature of CV dye in solution. At lower pH values, the abundance of H + ions create competition with the positively charged dye molecules for the active hydroxyl sites (Si-OH and Al-OH) on the diatomite surface, thereby reducing adsorption efficiency^55^. Conversely, as pH increases, H + ion concentration diminishes while HO- ion concentration rises^56^, facilitating the proposed mechanism illustrated in Equations (i) and (ii).

i $$\:\text{S}\text{i}-\text{O}\text{H}+{\text{H}\text{O}}^{-}{\leftrightarrow\:\text{S}\text{i}-\text{O}}^{-}+{\text{H}}_{2}\text{O}$$

ii $$\:{\text{S}\text{i}-\text{O}}^{-}+{\text{C}\text{V}}^{+}{\leftrightarrow\:\text{S}\text{i}-\text{O}}^{-+}\text{C}\text{V}$$

The effect of pH on CV sorption can also be explained by determining the pH_pzc_ values of the adsorbents, which indicate the ability of the adsorbent surface to become protonated (positively charged) or deprotonated (negatively charged)^57^. Figure 5(b) shows that the zero point of charge for RD is $\:\sim\:$5.4 and for DT is $\:\sim$ 6.1. Thus, at pH values above the pH_pzc_, the surface of the adsorbent becomes negatively charged, enhancing the electrostatic attraction between the surface and the positively charged CV dye molecules, leading to increased adsorption. In contrast, at pH levels below the pH_pzc_, the surface is more positively charged, creating electrostatic repulsion between the adsorbent and the CV dye, which reduces adsorption efficiency.

#### Effect of contact time

**Fig. 6:**
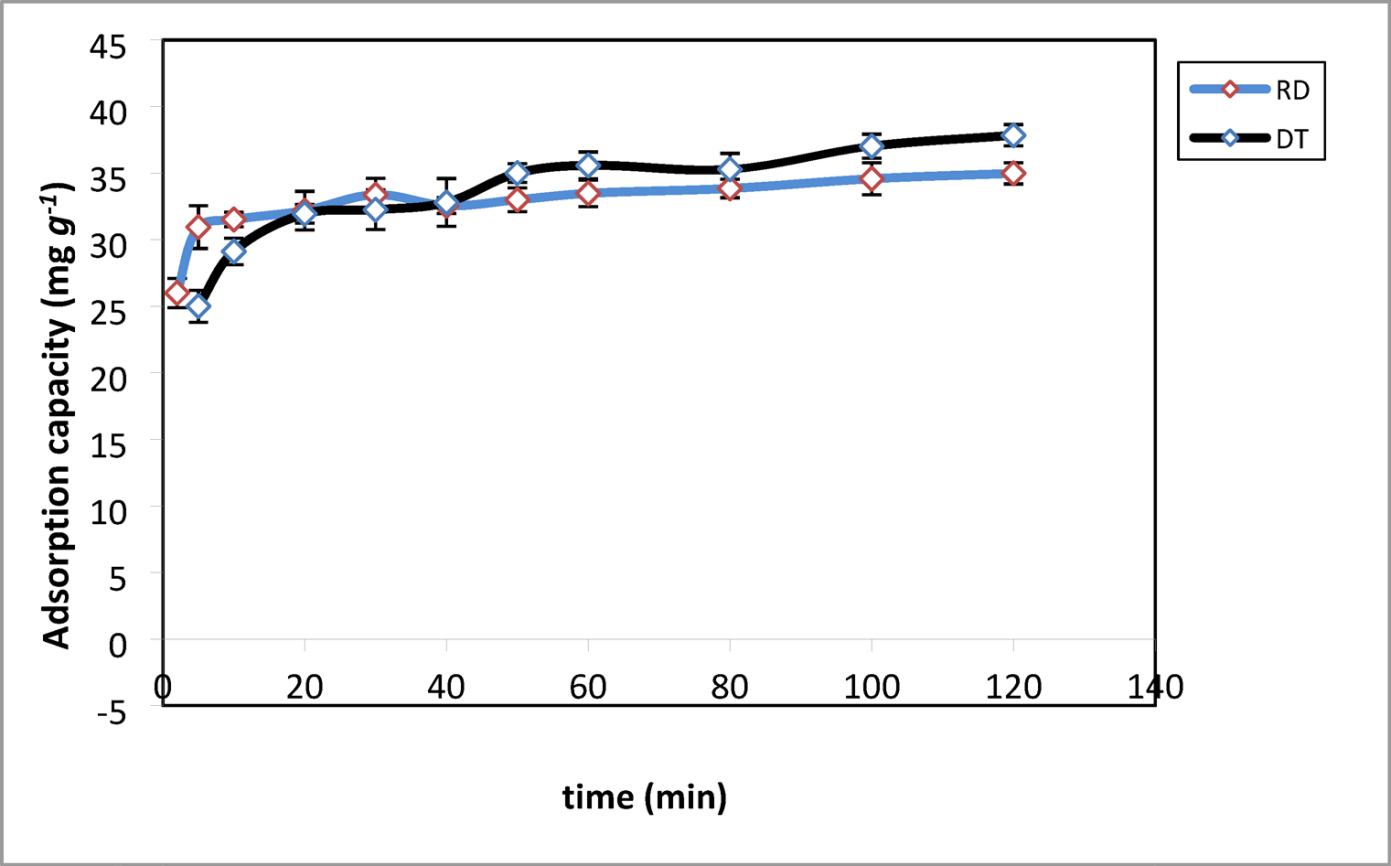
Effect of contact time on the adsorption of CV dye. Experimental conditions: C_0_ = 40 mg. g^− 1^, m = 50 mg, T = 25 °C, pH (DT = 8, RD = 10).

The determination of the effects of contact time is a key factor in sorption studies as it gives valuable information on the length of time needed for the adsorption process of CV an adsorbate-adsorbent system to equilibrate^58^. This in turn helps in the designing and planning of the removal of CV from aqueous solution. Figure 6; shows the effect of contact time on the adsorption process of CV on DT and RD. It was observed that a contact time of about 60 min was found to be sufficient to achieve adsorption equilibrium. However, 120 min was chosen as the best contact period for all tests. In the beginning of the adsorption process of CV on DT and RD from aqueous solution, it was observed that the adsorption rate of CV on both adsorbents accelerated with increasing time. Thus, it was rapid for the first 10 min this is due to the CV molecules interacting quickly with plenty of available active sites on the external surface of DT and RD. And then gradually reached equilibrium between 60 and 120 min. This is because nearly all the available active sites are occupied by CV and that the adsorption capacity tends to remain steady^59^. As a result, the maximum uptakes of CV on the DT and RD adsorbents from aqueous solutions were found as 38 mg.g^− 1^ and 34.98 mg.g^− 1^, respectively.

#### Effect of adsorbent dose

**Fig. 7:**
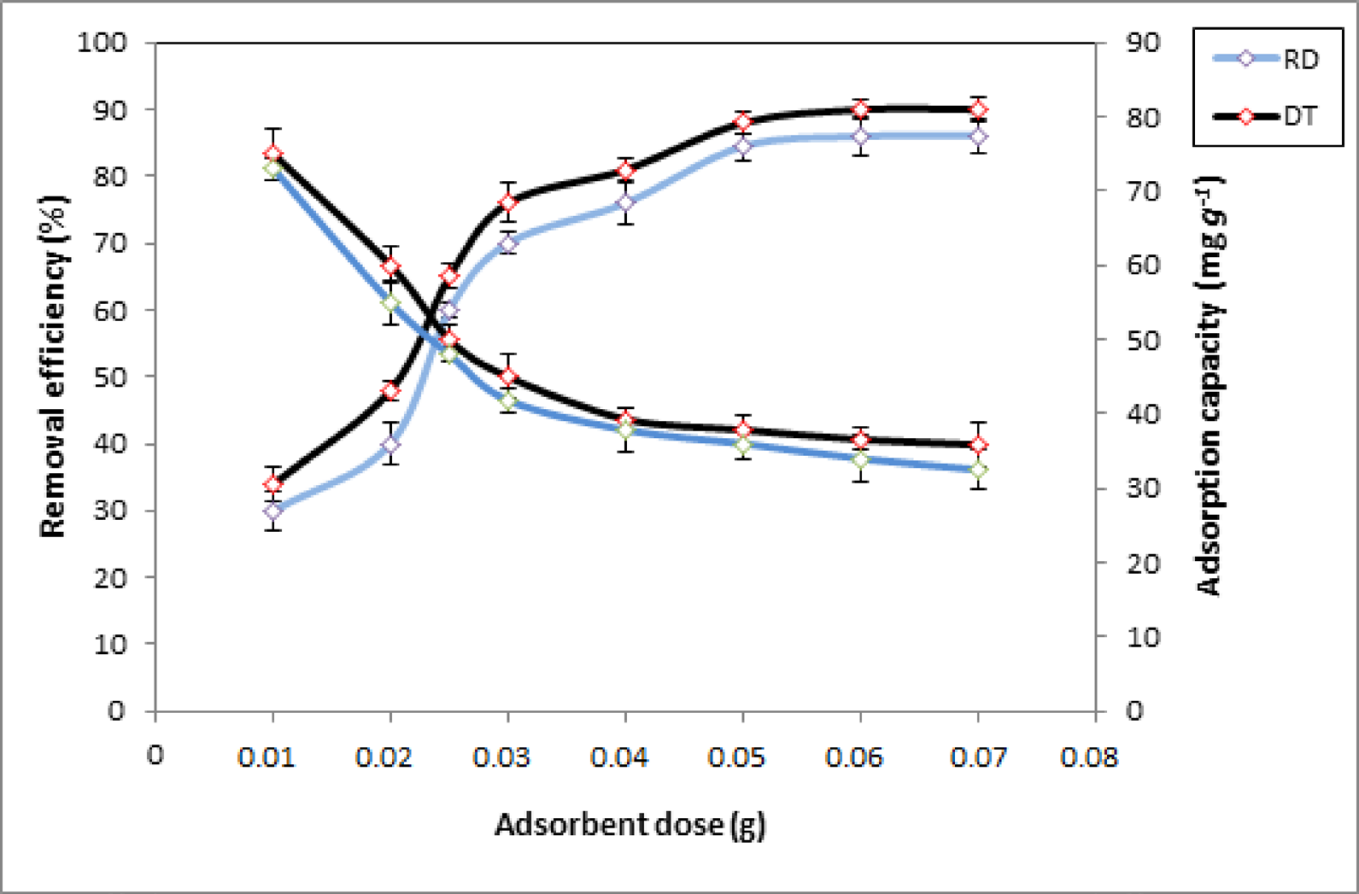
Effect of the adsorbent dose on the adsorption of CV dye. C_0_ = 40 mg. g^− 1^, t = 120 min, T = 25 °C, pH (DT = 8, RD = 10).

The adsorption of CV on DT and RD is studied by changing the quantity of adsorbent from 0.01 to 0.07 g in a 50 mL solution of 40 mg.L^− 1^ dye concentration at a constant stirring rate of 120 min. Figure 7 shows that the removal efficiency of the dye increases from 34.17 to 90.03% for DT and from 30 to 86.12% for RD as the adsorbent dose has increased from 0.01 to 0.07 g. This is due to the increased number of adsorption sites made available by increasing the adsorbent dose^60^. On the other hand, when the adsorbent dose increased from 0.01 to 0.07 g, the adsorption capacity decreased for both adsorbent from 75.02 mg.g^− 1^ to 35.83 mg.g^− 1^ and 73mg.g^− 1^ to 32.55 mg.g^− 1^ for DT and RD respectively. The reason for this is that at high adsorbent doses, the available dye molecules are insufficient to completely cover the adsorbent’s accessible binding sites^61^. Conversely, the observed reduction in removal efficiency at higher adsorbent dosages can be attributed to the establishment of equilibrium between available active sites on the adsorbent surface and the concentration of dye molecules in the solution^62^. As a result, increasing the adsorbent dose did not improve removal efficiency. Finally, for mass 0.05 g per 50 mL of dye solution, the optimal adsorption performance of CV on both adsorbent materials was determined as a removal percentage value of 89% and 85.2% for DT and RD, respectively.

#### Effect of initial dye concentration

**Fig. 8:**
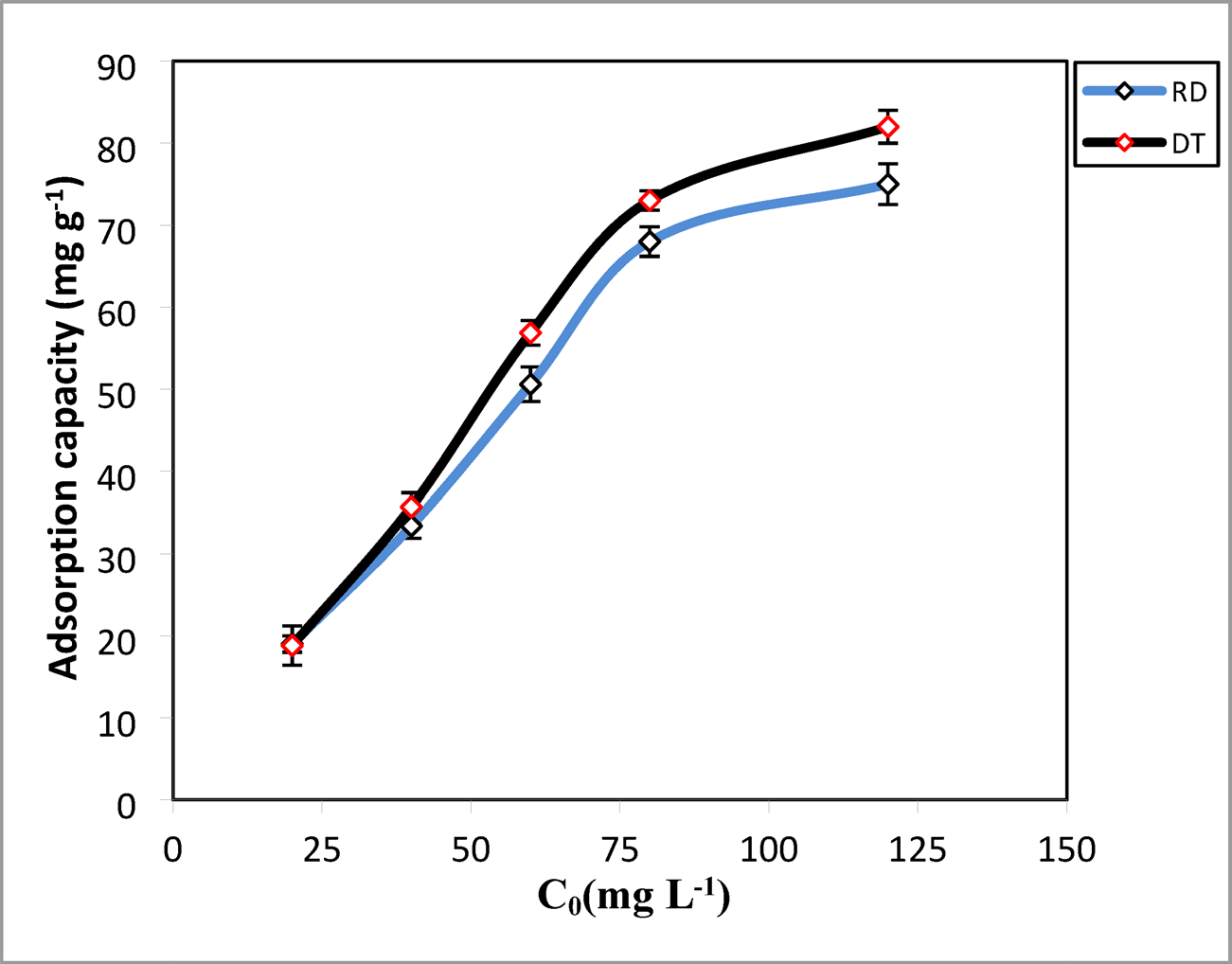
Effect of initial CV concentration on the adsorption capacity. m = 50 mg, t = 120 min, T = 25 °C, pH (DT = 8, RD = 10).

The impact of initial CV concentration on the uptake capacity was assessed from 20 mg. L^− 1^ to120 mg L^− 1^, with equilibrium achieved within 120 min, as shown in Fig. 8. This figure showed the effect of the initial concentration of CV adsorption varying from 20 to 120 mg. L^− 1^ on both adsorbents. When the initial concentration of CV increased from 20 mg. L^− 1^ to 120 mg.L^− 1^, the adsorption amount increased from 18.80 mg. g^− 1^ to 82 mg.g^− 1^ and 19 mg.g^− 1^ to 75 mg.g^− 1^ for DT and RD, respectively. This can be explained as more surface area and vacant adsorption sites were available for CV^63^. On the other hand, this increase is attributed to the availability of internal and external active sites on the surface of the adsorbent that have not yet been completely occupied by the dye^64^.

#### Temperature effect

**Fig. 9:**
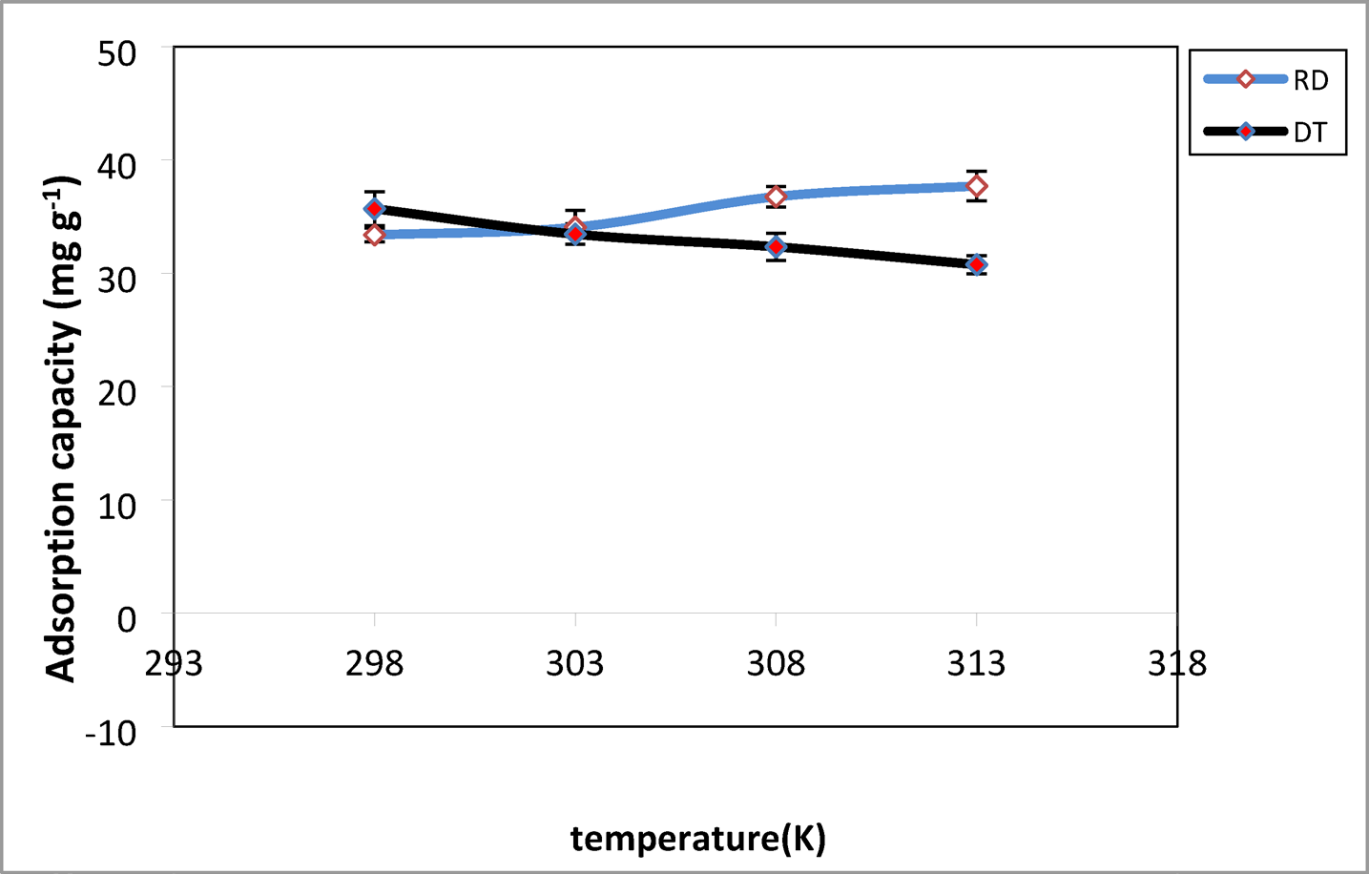
Effect of temperature on the adsorption of CV dye. C_0_ = 40 mg. g^− 1^, m = 50 mg, pH (DT = 8, RD = 10), t = 120 min.

Temperature has a significant impact on the structure and properties of the adsorbent^63^. The effect of temperature (298–313 K) on CV adsorption capacity was examined, with equilibrium achieved within 120 min. As shown in Fig. 9 the adsorption capacity has decreased from 36 to 30.77 mg.g^− 1^ for DT and has increased from 33.38 to 37.7 mg. g^− 1^ for RD as the temperature has increased from 298 K to 313 K. Consequently, the values of the greatest adsorption capacity of CV dye were seen at 298 K and 313 K for DT and RD, respectively. In other words, no special temperature was required for CV adsorption onto both adsorbent materials. As a result, the adsorption process was exothermic for DT and endothermic for RD.

### Kinetics study

**Fig. 10:**
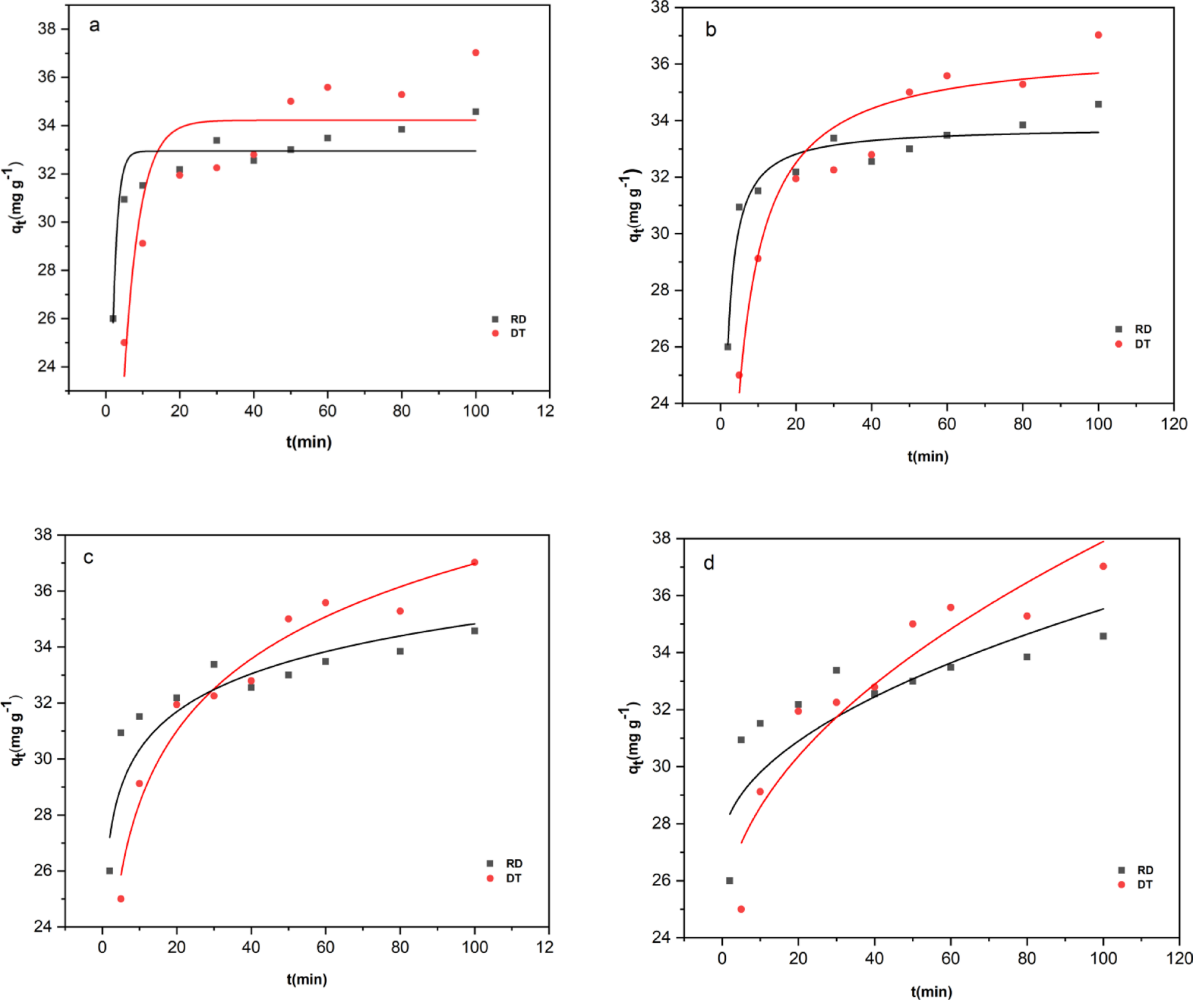
Nonlinear fitting to pseudo-first-order (**a**), pseudo-second-order (**b**), Elovich (**c**), and Intraparticle diffusion (**d**) models.

Kinetic studies are used to analyze the behavior of the transfer of pollutants on the sorbent surface per unit of time or effective variables on the rate of sorption process. In this study, linear and nonlinear pseudo-first-order^65^, pseudo-second-order^66^, Elovich^67^, and intraparticle diffusion models^68^, were used to investigate the kinetic behavior of CV dye sorption using raw and treated diatomite. The equations for these models are presented in Sect. 2.6. The nonlinear plots are presented in Fig. 10, and the results of the kinetic models, as reported in Table 2. The value of correlation coefficient (R^2^) of pseudo-second-order for both adsorbents is $\:\sim$ 1, while the corresponding R² values for the pseudo-first-order, intraparticle diffusion, and the Elovich kinetic models are lower than 1. In meantime the adsorption capacity q_e, cal_ (mg. g^−1^) calculated by the PSO model is also close to those determined by experiments q_e_,_exp_(mg. g^− 1^) for both adsorbent. These results suggest that the PSO model is more appropriate for describing the adsorption kinetics of CV dye. The Elovich model, which describes chemisorption on heterogeneous surfaces^67^, showed lower R² values compared to the PSO model, indicating a less accurate fit. However, the high α value for RD suggests a rapid initial adsorption rate, while the higher β value implies a faster decline in adsorption over time^68^. This suggests that although chemisorption is involved, the Elovich model is less suitable than the PSO model for describing CV dye adsorption on both DT and RD. Meanwhile, the intraparticle diffusion model helps to assess whether pore diffusion influences the adsorption process^68^. With lower R² values for both adsorbents, suggests that the intraparticle diffusion plots do not pass through the origin, suggesting that while intraparticle diffusion occurs, it is not the only rate-limiting step in the adsorption of CV dye^69^.

**Table 2:** Kinetic parameters for CV dye adsorption onto diatomite DT and RD.

Kinetic models	parameter	adsorbents	
		DT	RD
Pseudo-first order	R^2^	0.764	0.841
	q_e, cal_(mg. g^− 1^)	34.2	32.9
	k_1_ (min^− 1^)	0.234	0.754
	q_e, exp_ (mg. g^− 1^)	38.0	34.9
Pseudo-second order	R^2^	0.998	0.999
	q_e, cal_ (mg. g^− 1^)	40.0	34.8
	k_2_(g/ mg. min)	0.0051	0.023
	q_e, exp_(mg. g^− 1^)	38.0	34.9
Intraparticle diffusion	R^2^	0.889	0.693
	C (mg/g)	24.3	28.1
	k_id_ (mg/(g·min^0.5^))	1.36	0.703
Elovich	R^2^	0.963	0.849
	α (mg/g.min)	781	7.04 $\:\times\:$10^6^
	β (g/mg)	0.269	0.572

### Adsorption isotherm

**Fig. 11:**
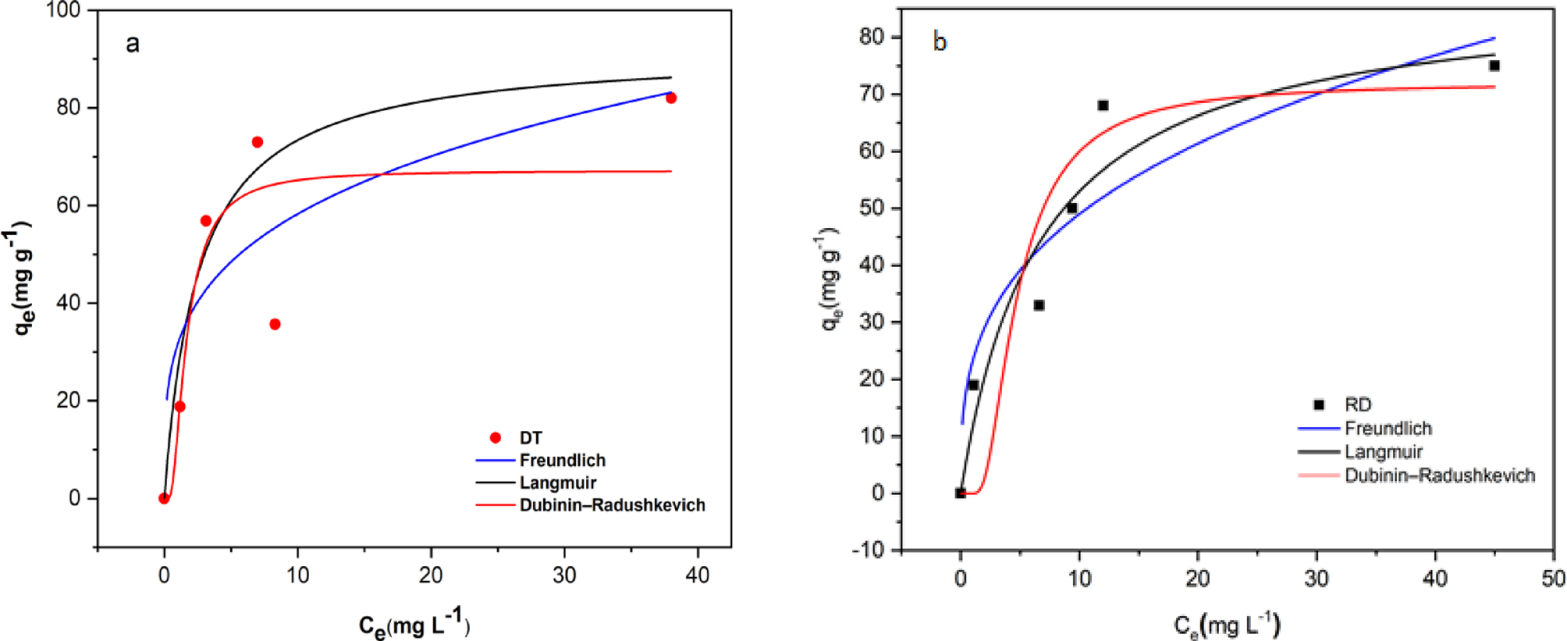
Non-linear fitting to Langmuir, Freundlich, and Dubinin–Radushkevich models of the adsorptions of CV onto diatomite (a: DT, b: RD).

The experimental adsorption curves of the present work were adjusted to the Langmuir^70^, Freundlich^71^, and Dubinin–Radushkevich (D–R) models^72^ The equations for these models are presented in Sect. 2.6. For the Langmuir model, the favorable nature of adsorption can be expressed in terms of the dimensionless separation factor of the equilibrium parameter (RL), as defined by Eq. 10 in the Sect. 2.6. The adsorption is irreversible if RL = 0, If 0 < RL < 1, the CV dye molecules can be easily adsorbed. at RL = 1, the adsorption process is linear, and if RL > 1, the CV dye adsorption is difficult to occur^73^. In the Freundlich Adsorption equation, the adsorption process will be linear (*n* < 1) or chemical (*n* = 1) or physical (*n* > 1), the slope ranges between 0 and 1 is a measure of sorption intensity, becoming more nonhomogeneous when its value gets closer to zero^73^. Another parameter to determine the type of adsorption process (physical or chemical) is the mean free energy, E, which can be calculated from the D-R isotherm model (Eq. 11, Sect. 2.6). Accordingly, for (E < 8) and (8 < *E* < 16) kJ/mol, the adsorption process will be physical and chemical, respectively^74^. When the adsorption data obtained at the equilibrium adsorption concentration were applied to the Langmuir, Freundlich, and D-R isotherm models, the nonlinear curves were obtained Fig. 11(a, b) and the isotherm parameters were calculated as seen in Table 3, it can be pronouncedly viewed that the Langmuir model is the best-fit model for the adsorption of CV on both adsorbents with correlation coefficient R^2^ value of (0.960 for DT and 0.929 for RD) meanwhile the R^2^ came from Freundlich, and D-R isotherm models depict a low fitting model with lower regression coefficients (0.561 for DT and 0.815 for RD) and (0.771 for DT and 0.891 for RD) respectively which gives a signal that the interaction of sorbate and sorbent may not fully follow these models. The value of *R*^2^ makes the Langmuir model most suitable to describe the adsorption process and indicate that the CV adsorption process on both adsorbents due to homogeneous monolayer coverage and the adsorption sites are independent of each other^75^. The maximum amount of CV adsorption was calculated at 91.95 mg. g^− 1^ for DT and 88.36 mg. g^− 1^ for RD. The RL value between 0 and 1 (0.020-0,112) for DT and (0.052–0.251) for RD, signified that the CV dye molecules are easily adsorbed on the active sites of adsorbents in other hand the adsorption process of CV is considered favorable^75^. The Dubinin–Radushkevich (D–R) isotherm model was also used to identify the type of adsorption. The calculated energy values (E) were 0.389 kJ/mol for DT and 0.157 kJ/mol for RD. Since both values are less than 8 kJ/mol, this confirms that the CV adsorption onto diatomite is a physical process, involving weak interactions like van der Waals forces^74^.

**Table 3:** The parameters of langmuir, freundlich, and Dubinin–Radushkevich models for the adsorption of CV onto diatomite (DT, RD).

	Langmuir			Freundlich			Dubinin–Radushkevich		
	q_max_(mg. g^− 1^)	K_L_ (L/g )	*R* ^2^	K_F_ (mg^1s − *n*^g-^1^L^−*n*^)	1/*n*	*R* ^2^	q_m_ (mg. g^− 1^)	E kJ/mol	*R* ^2^
DT	91.9	0.395	0.960	31.6	0.266	0.561	67.2	0.389	0.771
**RD**	**88.4**	**0.149**	**0.929**	**23.2**	**0.324**	**0.815**	**72.0**	**0.157**	**0.891**

Table 4 shows the maximum adsorption capacities of CV from aqueous solutions using various adsorbents. The higher adsorption capacities of certain materials can be attributed to their structural and morphological properties, such as high surface area, pore size distribution, and specific experimental conditions. In this study, the adsorption capacity of treated diatomite (82 mg·g⁻¹) was significantly higher than other adsorbents reported in the literature. This enhanced performance resulted from the acid treatment, which substantially increased the surface area and porosity of the diatomite. Additionally, the removal of carbonate impurities and the increased number of silanol groups strengthened the electrostatic interactions between the adsorbent and the cationic CV dye molecules.

**Table 4:** Comparison of the adsorption capacities of some adsorbents for removal of CV.

Adsorbent	Contact time (min)	Adsorbent dose (g)	q_max_ (mg. g^− 1^)	Ref.
Moroccan pyrophyllite	60	0.10	9.58	^78^
Activated carbon	90	0.05	31.24	^79^
Polyvinyl alcohol/agar/maltodextrin	120	0.02	19.17	^80^
Modified almond shell	180	0.10	12.2	^81^
Olive leave powder (OLP)	60	0.05	12.32	^82^
Biochar from palm kernel shell (BC-PKS)	60	0.10	24.45	^83^
Maleic anhydride-modified cellulose fibers/diatomite	120	0.05	61.1	^84^
Kaolin	150	0.05	31.94	^85^
RBW-III	120	0.05	30.58	^86^
β-cyclodextrin onto mesoporous silica	180	0.05	37.5	^87^
White potato peel powder	60	0.05	17.13	^88^
Bio-Hap	120	0.05	37.93	^89^
Raw alluvium	90	0.05	57.84	^90^
Raw diatomite	120	0.05	75	Present work
Treated diatomite	120	0.05	82	Present work

### Thermodynamics studies

**Fig. 12:**
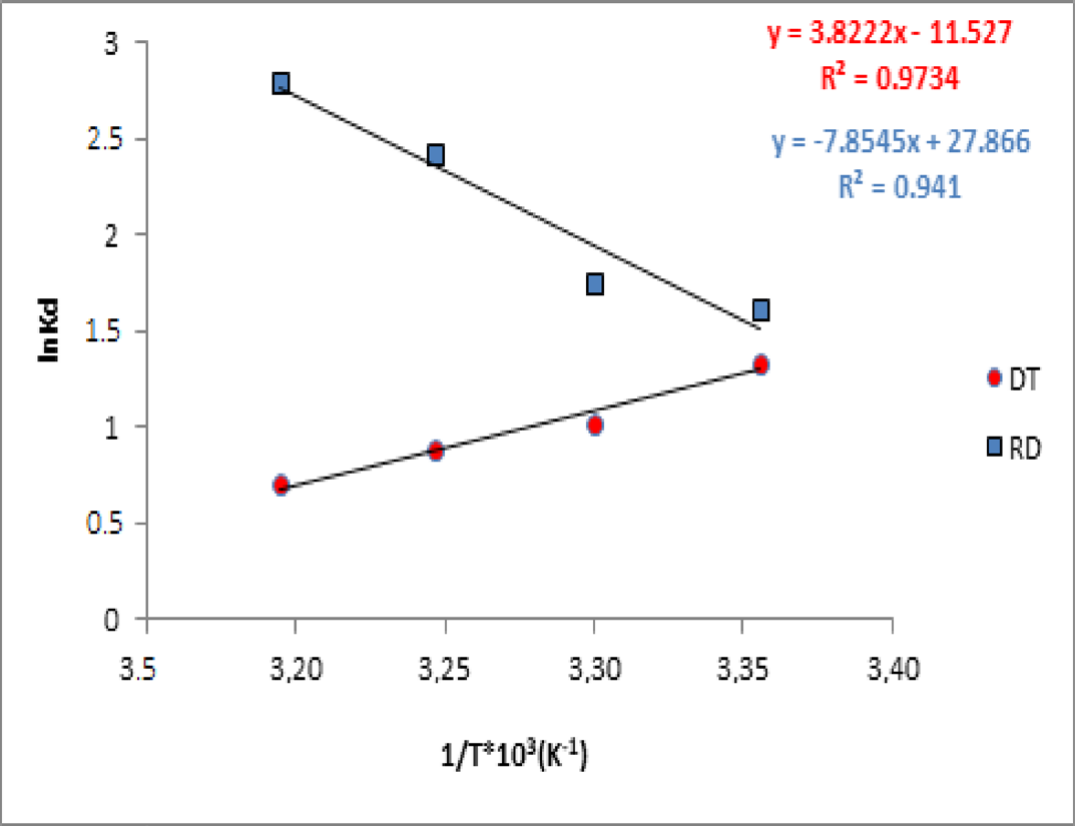
Thermodynamics of the adsorption of the CV dye onto diatomite (DT, RD).

Thermodynamic parameters were determined to investigate the temperature effect on the adsorption. Hence, Gibbs free energy change ($\:\varDelta\:G^\circ\:$), standard enthalpy change ($\:\varDelta\:H^\circ\:$), and standard entropy change ($\:\varDelta\:S^\circ\:$) were calculated using the Van’t Hoff equations (Eqs. 12, 13, and 14 in Sect. 2.6)^73^. The thermodynamic parameters *ΔH°* and *ΔS°* were calculated from the slopes and intercepts for the linear variation of lnKd versus 1/T by Eq. (14). The values of thermodynamic parameters such as ∆H^o^, ∆S^o^ and ∆G^o^ were determined and the. results presented in Fig. 12; Table 5, indicate that adsorption onto DT is exothermic (ΔH° < 0) and accompanied by a reduction in the randomness of the solid/liquid (ΔS° < 0), whereas adsorption onto RD is endothermic (ΔH° > 0) and there is affinity of RD for the dye molecules with an increase in randomness of the solid/liquid interface (ΔS° > 0). In both cases, the negative ΔG° values confirm the spontaneity of the process.

These findings can be explained by the surface properties of the adsorbents before and after acid treatment. For DT, acid treatment increased the SiO_2_ content and enhanced surface negativity, promoting exothermic crystal violet binding via electrostatic interactions. The alignment of dye molecules on the relatively homogeneous silanol sites led to a decrease in interfacial disorder. In contrast, RD possesses a heterogeneous surface containing carbonates and metal oxides, which required additional energy to displace hydrated ions and accommodate the dye molecules. This resulted in increased disorder and endothermic adsorption behavior^76,77^.

**Table 5:** Thermodynamic parameters of the adsorption of the CV dye onto diatomite (DT, RD).

	ΔH°	ΔS°	ΔG° (kJ.mol^− 1^)				R^2^
	(J.mol^− 1^)	(J.mol^− 1^ K^− 1^)	298 K	303 K	308 K	313 K	
DT	-31.77	-95.83	-3.3175	-2.5849	-2.2721	-1.8311	0.973
**RD**	**65.3**	**231.67**	**-4.0083**	**-4.3994**	**-6.2195**	**-7.2779**	**0.941**

## Conclusion

This study investigated the potential of low-cost adsorbents, raw and treated diatomite, for removing CV from aqueous solutions. Various characterization techniques, including SEM, XRD, FTIR, BET, GTA, pH_pzc_ and XRF, were employed to assess the adsorbents’ properties. The adsorption process was analyzed by considering factors such as contact time, pH, adsorbent dosage, initial dye concentration, and temperature. Under optimal conditions pH 8 for DT and pH 10 for RD, initial dye concentration of 120 mg.L^− 1^, contact time of 120 min, adsorbent dose of 0.05 mg, and temperature of 298 K—the maximum adsorption capacities achieved were 82 mg.g^− 1^ for treated diatomite and 75 mg.g^− 1^ for raw diatomite. Kinetic studies indicated that the adsorption followed the pseudo-second-order model. The Langmuir model provided the best fit for the adsorption isotherm. Thermodynamic analysis revealed that the adsorption onto DT was exothermic, accompanied by a reduction in the randomness of the solid/liquid interface. Conversely, adsorption onto RD was endothermic, leading to an increase in interface randomness. Negative values of ΔG° indicated the spontaneity of the adsorption process for both adsorbents. These findings underscore the potential of raw and treated diatomite as effective and economical adsorbents for the removal of CV. Further research can focus on optimizing the adsorption process parameters, exploring scalable implementation, investigating effective regeneration strategies to evaluate the reusability of the spent adsorbents, and assessing the efficacy for removing other cationic dyes. These endeavors will contribute to the practical application of raw and treated diatomite as sustainable adsorbents for water treatment and environmental remediation.

## Electronic supplementary material

Below is the link to the electronic supplementary material.

Supplementary Material 1

Supplementary Material 2

## Data Availability

The XRD datasets generated and/or analyzed during the current study are available in the Research Unit on Emerging Materials (RUEM) at https://www.univ-setif.dz/.

## References

[1] Singh, V. *Water Pollution. Textbook of Environment and Ecology*253–266 (Springer Nature, 2024).

[2] Zenati, B. et al. Pollutant load discharge from a Southwestern mediterranean river (Mazafran river, Algeria) and its impact on the coastal environment. *Arab. J. Geosci.* **16** (3), 146. 10.1007/s12517-023-11260-0 (2023).

[3] Sharma, J., Sharma, S. & Soni, V. Classification and impact of synthetic textile dyes on aquatic flora: A review. *Reg. Stud. Mar. Sci.* **45**, 101802. 10.1016/j.rsma.2021.101802 (2021).

[4] Al-Tohamy, R. et al. A critical review on the treatment of Dye-Containing wastewater: ecotoxicological and health concerns of textile dyes and possible remediation approaches for environmental safety. *Ecotoxicol. Environ. Saf.* **231**, 113160. 10.1016/j.ecoenv.2021.113160 (2022). 35026583 10.1016/j.ecoenv.2021.113160

[5] Uddin, M. K. & Nasar, A. et Decolorization of basic dyes solution by utilizing fruit seed powder *KSCE J. Civ. Eng.*, **24**, 2 345–355 (2020). 10.1007/s12205-020-0523-2

[6] Koyuncu, I., Topacik, D. & Yuksel, E. Reuse of reactive dyehouse wastewater by nanofiltration: process water quality and economical implications. *Sep. Purif. Technol.* **36** (1), 77–85. 10.1016/S1383-5866(03)00154-0 (2004).

[7] Ingrao, C. et al. Water scarcity in agriculture: An overview of causes, impacts and approaches for reducing the risks. Heliyon (2023). 10.1016/j.heliyon.2023.e18507 PMC10392093 37534016

[8] Hübner, U. et al. Advanced oxidation processes for water and wastewater treatment–Guidance for systematic future research. Heliyon (2024). 10.1016/j.heliyon.2024.e30402 PMC11079112 38726145

[9] Uddin, M. K., Mashkoor, F., AlArifi, I. M. & Nasar, A. et Simple one-step synthesis process of novel MoS2@bentonite magnetic nanocomposite for efficient adsorption of crystal violet from aqueous solution,* Mater. Res. Bull.*** 139**, 111279, (2021). 10.1016/j.materresbull.2021.111279

[10] Chauhan, K. et al. Photo-catalytic removal of Rhodamine B by nickel doped graphitic carbon nitride: anomalous dependence of removal efficiency on carrier recombination. *J. Iran. Chem. Soc.* 1–18. (2024).

[11] Tran, Q. et al. Hydrate technology for water desalination in the Mekong Delta. *Vietnam Heliyon*. **10**, 19 (2024). 10.1016/j.heliyon.2024.e38974 PMC11491894 39435117

[12] Katheresan, V., Kansedo, J. & Lau, S. Y. Efficiency of various recent wastewater dye removal methods: A review. *J. Environ. Chem. Eng.* **6** (4), 4676–4697. 10.1016/j.jece.2018.06.060 (2018).

[13] Saravanan, A. et al. Effective water/wastewater treatment methodologies for toxic pollutants removal: processes and applications towards sustainable development.* Chemosphere*** 280**, 130595. (2021).10.1016/j.chemosphere.2021.130595 10.1016/j.chemosphere.2021.130595 33940449

[14] Qasem, N. A. A., Mohammed, R. H. & Lawal, D. U. Removal of heavy metal ions from wastewater: A comprehensive and critical review. *Npj Clean. Water*. **4** (1), 1–15. 10.1038/s41545-021-00127-0 (2021).

[15] Singh, N. B., Nagpal, G., Agrawal, S. & Rachna Water purification by using adsorbents: A review. *Environ. Technol. Innov.* **11**, 187–240. 10.1016/j.eti.2018.05.006 (2018).

[16] Martin, M. J., Artola, A., Balaguer, M. D. & Rigola, M. Activated carbons developed from surplus sewage sludge for the removal of dyes from dilute aqueous solutions. *Chem. Eng. J.* **94** (3), 231–239. 10.1016/S1385-8947(03)00054-8 (2003).

[17] Desorption- and decomposition‐based techniques for the regeneration of activated carbon. * Chemical Engineering & Technology* . Wiley Online Library. https://onlinelibrary.wiley.com/doi/abs/10.1002/ceat.201300808 (accessed 2023-06-26).

[18] Awual, M. R., Hasan, M. M. & Shahat, A. Functionalized novel mesoporous adsorbent for selective Lead(II) ions monitoring and removal from wastewater. *Sens. Actuators B*. **203**, 854–863. 10.1016/j.snb.2014.07.063 (2014).

[19] Indhumathi, P. et al. The efficient removal of heavy metal ions from industry effluents using waste biomass as Low-Cost adsorbent: thermodynamic and kinetic models. *Z. FÃ¼r Phys. Chem.* **232** (4), 527–543. 10.1515/zpch-2016-0900 (2018).

[20] Wang, B. et al. Development of nanocomposite adsorbents for heavy metal removal from wastewater. *ES Mater. Manuf.* **2** (5), 35–44 (2018).

[21] Nargis, F., Duong, A., Rehl, E., Bradshaw, C. & Kazemian, H. Highly efficient and Low-Cost Clay-Based adsorbent for glyphosate removal from contaminated water. *Chem. Eng. Technol.* **45** (2), 340–347. 10.1002/ceat.202100437 (2022).

[22] Ugwu, E. I., Othmani, A. & Nnaji, C. C. A review on zeolites as Cost-Effective adsorbents for removal of heavy metals from aqueous environment. *Int. J. Environ. Sci. Technol.* **19** (8), 8061–8084. 10.1007/s13762-021-03560-3 (2022).

[23] Varsha, M., Senthil Kumar, P. & Senthil Rathi, B. A. Review on recent trends in the removal of emerging contaminants from aquatic environment using Low-Cost adsorbents. *Chemosphere* **287**, 132270. 10.1016/j.chemosphere.2021.132270 (2022). 34560497 10.1016/j.chemosphere.2021.132270

[24] Kashin, A. D. et al. Diatomite-Based ceramic Biocoating for magnesium implants. *Ceram. Int.* **48** (19, Part A), 28059–28071. 10.1016/j.ceramint.2022.06.111 (2022).

[25] Diatom biosilica. Source, physical-chemical characterization, modification, and application - Saoud – 2022 - Journal of Separation Science - Wiley Online Library. https://analyticalsciencejournals.onlinelibrary.wiley.com/doi/abs/10.1002/jssc.202100981 (accessed 2023-06-26). 10.1002/jssc.202100981 35652201

[26] Łach, M. et al. Use of diatomite from Polish fields in sustainable development as a sorbent for petroleum substances. *J. Clean. Prod.* **389**, 136100. 10.1016/j.jclepro.2023.136100 (2023).

[27] Han, L. et al. Superhydrophilic/air-superoleophobic diatomite porous ceramics for highly-efficient separation of oil-in-water emulsion. *J. Environ. Chem. Eng.*, **10** (5), 108483 (2022).10.1016/j.jece.2022.108483

[28] Touina, A. et al. Characterization and efficient dye discoloration of Algerian diatomite from Ouled Djilali-Mostaganem. *SN Appl. Sci.* **3** (4), 476. 10.1007/s42452-021-04334-9 (2021).

[29] Li, X. et al. Optimization of Diatom-Based blotting materials and their efficient selective adsorption of Pb(II). *Mater. Today Commun.* **36**, 106434. 10.1016/j.mtcomm.2023.106434 (2023).

[30] Li, M. et al. Efficient removal of Cd2 + by diatom frustules Self-Modified in situ with intercellular organic components. *Environ. Pollut.* **319**, 121005. 10.1016/j.envpol.2023.121005 (2023). 36608731 10.1016/j.envpol.2023.121005

[31] Benhalima, T., Allali, F. Z., Roumane, N. & Ferfera-Harrar, H. Enhanced adsorptive removal of hazardous Methyl Violet 2B and Methyl orange dyes by Algerian Diatomite-Loaded Polysaccharide-Based hydrogel beads. *J. Mol. Liquids*, **383**, 122150. (2023).10.1016/j.molliq.2023.122150

[32] Enhanced methylene blue adsorption from aqueous solution by corn stalk/diatomite gel porous materials. Journal of Dispersion Science and Technology: Vol 0, No 0. (2023). https://www.tandfonline.com/doi/abs/10.1080/01932691.2195928 (accessed 2023-07-02).

[33] Radjai, M. et al. Adsorptive removal of cationic and anionic dyes on a novel mesoporous adsorbent prepared from diatomite and anionic cellulose nanofibrils: experimental and theoretical investigations. *J. Mol. Liq.* **361**, 119670. 10.1016/j.molliq.119670 (2022).

[34] Ouallal, H. et al. Study of acid treatment effect of a natural red clay onto physico-chemical and adsorption properties. *Desalination Water Treat.* **315**, 96–110 (2023).

[35] Afroze, S. & Sen, T. K. A review on heavy metal ions and dye adsorption from water by agricultural solid waste adsorbents. *Water Air Soil. Pollut*. **229** (7), 225. 10.1007/s11270-018-3869-z (2018).

[36] Kim, D. et al. High-Performance adsorbent for ethane/ethylene separation selected through the computational screening of Aluminum-Based Metal–Organic frameworks. *ACS Appl. Mater. Interfaces*. **14** (38), 43637–43645. 10.1021/acsami.2c13905 (2022). 36124874 10.1021/acsami.2c13905

[37] Sall, S. et al. Diatom and diatomite: different focus on natural media to material science path. *Am. J. Anal. Chem.* **15** (01), 1–29. 10.4236/ajac.2024.151001 (2024).

[38] Dehmani, Y. et al. Comparison of phenol adsorption property and mechanism onto different Moroccan clays. *Water* **15** (10), 1881 (2023).

[39] Arfaoui-Elhif, R. et al. Development by emulsion templating of a novel Tunisian clay-polyvinyl alcohol/extra-virgin Olive oil scaffold with antibiofilm properties. *Colloids Surf., A*. **677**, 132421 (2023).

[40] Adazabra, A. N. et al. Valorising cassava pomace biosolid in fired clay bricks production: physical, mechanical and thermal evaluation. *Mater. Chem. Phys.* **309**, 128402 (2023).

[41] Pan, X. et al. Characteristic, purification and application of quartz: A review. *Miner. Eng.* **183**, 107600. 10.1016/j.mineng.2022.107600 (2022).

[42] Gao, R., Liu, D., Huang, Y. & Li, G. Preparation of Diatomite-Modified wood ceramics and the adsorption kinetics of Tetracycline. *Ceram. Int.* **46** (12), 19799–19806. 10.1016/j.ceramint.2020.05.014 (2020).

[43] Adsorption behavior of Janus Green B dye on Algerian diatomite - IOPscience. https://iopscience.iop.org/article/10.1088/2053-1591/ab2732/meta (accessed 2023-07-09).

[44] Baba, F., Benaliouche, F., Meknaci, R. & Boucheffa, Y. Water adsorption and antibacterial activity studies for characterization of Ca-LTA zeolite/diatomite adsorbents. *Colloid Interface Sci. Commun.* **35**, 100233. 10.1016/j.colcom.2020.100233 (2020).

[45] Mohamed, E. A. et al. Enhancing adsorption capacity of Egyptian diatomaceous Earth by Thermo-Chemical purification: methylene blue uptake. *J. Colloid Interface Sci.* **534**, 408–419. 10.1016/j.jcis.2018.09.024 (2019). 30245338 10.1016/j.jcis.2018.09.024

[46] Wang, S., Lee, Y. N., Nam, H., Nam, H. & Kim, H. K. Chemical activation of porous diatomite ceramic filter for the adsorption of TMA, H2S, CH3COOH and NH3: isotherm and kinetic studies. *J. Environ. Chem. Eng.* **7** (6), 103481. 10.1016/j.jece.2019.103481 (2019).

[47] Radev, L., Hristov, V., Michailova, I. & Samuneva, B. Sol-Gel bioactive Glass-Ceramics part I: calcium phosphate silicate/wollastonite Glass-Ceramics. *Open. Chem.* **7** (3), 317–321. 10.2478/s11532-009-0022-2 (2009).

[48] Silva, V. et al. Adsorption behavior of crystal Violet and congo red dyes on heat-treated Brazilian palygorskite: kinetic, isothermal and thermodynamic studies. *Materials* **14**, 5688 (2021). 34640085 10.3390/ma14195688 PMC8510337

[49] Alorabi, A. Q. et al. Natural clay as a low-cost adsorbent for crystal violet dye removal and antimicrobial activity.* Nanomaterials*** 11**11 ,2789. (2021). 10.3390/nano11112789 PMC8620351 34835556

[50] Wang, R. F. et al. Fabrication and characterization of sugarcane bagasse–calcium carbonate composite for the efficient removal of crystal Violet dye from wastewater. *Ceram. Int.* **46** (17), 27484–27492 (2020).

[51] Reka, A. A. et al. Diatomaceous earth: characterization, thermal modification, and application. *Open. Chemistry2021*, **19** (1), 451–461. 10.1515/chem-2020-0049

[52] Perederiy, I. & Papangelakis, V. G. Why amorphous FeO-SiO2 slags do not Acid-Leach at hightemperatures. *J. Hazard. Mater.* **321**, 737–744. 10.1016/j.jhazmat.2016.09.055 (2017). 27744239 10.1016/j.jhazmat.2016.09.055

[53] Sun, L. et al. Constructing nanostructured silicates on diatomite for Pb(II) and Cd(II) removal. *J. Mater. Sci.* **54** (9), 6882–6894. 10.1007/s10853-019-03388-w (2019).

[54] Liu, Y. et al. Degradation of Azo dyes with different functional groups in simulated wastewater by electrocoagulation. *Water* **14** (1), 123. 10.3390/w14010123 (2022).

[55] Yadav, S. et al. Adsorption of cationic dyes, drugs and metal from aqueous solutions using a polymer composite of Magnetic/β-Cyclodextrin/Activated charcoal/na alginate: isotherm, kinetics and regeneration studies. *J. Hazard. Mater.* **409**, 124840. 10.1016/j.jhazmat.2020.124840 (2021). 33482479 10.1016/j.jhazmat.2020.124840

[56] Synthesis of activated carbon from cherry tree waste. and its application in removing cationic red 14 dye from aqueous environments * Appl. Water Sci.*. https://link.springer.com/article/10.1007/s13201-023-01899-1 (accessed 2024-02-06).

[57] Missana, T. et al. Investigation of the surface charge behaviour of ettringite: Influence of pH, calcium, and sulphate ions.* Heliyon* 10.16 (2024). 10.1016/j.heliyon.2024.e36117 PMC11378879 39247325

[58] Kamal, M. H. M. A., Azira, W. M. K. W. K., Kasmawati, M., Haslizaidi, Z. & Saime, W. N. W. Sequestration of toxic Pb(II) ions by chemically treated rubber (Hevea Brasiliensis) leaf powder. *J. Environ. Sci.* **22** (2), 248–256. 10.1016/S1001-0742(09)60101-7 (2010). 10.1016/s1001-0742(09)60101-7 20397414

[59] The removal of methyl. Violet 2B dye using palm kernel activated carbon: thermodynamic and kinetics model . *Int. J. Environ. Sci. Technol.* https://link.springer.com/article/10.1007/s13762-019-02271-0 (accessed 2024-02-07).

[60] Utilization of chemically. Modified coal fly Ash as cost-effective adsorbent for removal of hazardous organic wastes. *Int. J. Environ. Sci. Technol.* https://link.springer.com/article/10.1007/s13762-022-04457-5 (accessed 2024-02-07).

[61] Removal of two. cationic dyes from aqueous solutions by adsorption onto local clay: experimental and theoretical study using DFT method: International Journal of Environmental Analytical Chemistry: Vol 103, No 6. (2021). https://www.tandfonline.com/doi/abs/10.1080/03067319.1873306 (accessed 2024-02-07).

[62] Waliullah, R. M. et al. Md. R. Optimization of toxic dye removal from contaminated water using Chitosan-Grafted novel nanocomposite adsorbent. *J. Mol. Liq.* **388**, 122763. 10.1016/j.molliq.2023.122763 (2023).

[63] Adsorption of methyl. violet from aqueous solution using β-cyclodextrin immobilised onto mesoporous silica:* Supramolecular Chemistry*** 33**, 4. (2021). https://www.tandfonline.com/doi/abs/10.1080/10610278.1917574 (accessed 2024-02-07).

[64] Adsorption Behavior and Mechanism of Methylene Blue, Violet, C. & Black, E. T, and Methyl orange dyes onto biochar-derived date palm fronds waste produced at different pyrolysis conditions* Water, Air, Soil Pollut.*. https://link.springer.com/article/10.1007/s11270-020-04595-x (accessed 2024-02-07).

[65] S., L. K About the theory of So-Called adsorption of soluble substances. *Sven Vetenskapsakad Handingarl*. **24**, 1–39 (1898).

[66] Ho, Y. S., Ng, J. C. Y. & McKay, G. Removal of Lead(Ii) from effluents by sorption on peat using Second-Order kinetics. *Sep. Sci. Technol.* **36** (2), 241–261. 10.1081/SS-100001077 (2001).

[67] Wu, F. C., Tseng, R. L. & Ruey-Shin, J. Characteristics of Elovich equation used for the analysis of adsorption kinetics in dye-chitosan systems. *Chem. Eng. J.* **150** (2-3), 366–373 (2009).

[68] Weber, W. J. Jr & Carrell Morris, J. Kinetics of adsorption on carbon from solution. *J. Sanit. Eng. Div.* **89** (2), 31–59 (1963).

[69] Suhaimi, N. et al. The use of gigantochloa bamboo-derived biochar for the removal of methylene blue from aqueous solution.* Adsorption Sci. Technol.* 8245797. (2022) (2022).

[70] Liu, Y. Some consideration on the Langmuir isotherm equation. *Colloids Surf., A*. **274** (1), 34–36. 10.1016/j.colsurfa.2005.08.029 (2006).

[71] Yang, C. & Statistical Mechanical Study on the Freundlich Isotherm Equation. *J. Colloid Interface Sci.* **208** (2), 379–387. 10.1006/jcis.1998.5843. (1998). 9845681 10.1006/jcis.1998.5843

[72] Theoretical basis for the potential theory adsorption isotherms. The Dubinin-Radushkevich and Dubinin-Astakhov equations Langmuir. https://pubs.acs.org/doi/pdf/10.1021/la00023a054 (accessed 2024-02-07).

[73] Dada, A. O., Olalekan, A., Olatunya, A. & Dada, O. Langmuir, freundlich, Temkin and Dubinin–Radushkevich isotherms studies of equilibrium sorption of Zn 2 + Unto phosphoric acid modified rice husk. *J. Appl. Chem.* **3**, 38–45. 10.9790/5736-0313845 (2012).

[74] Wang, J. & Guo, X. Adsorption isotherm models: classification, physical meaning, application and solving method. *Chemosphere* **258**, 127279. 10.1016/j.chemosphere.2020.127279 (2020). 32947678 10.1016/j.chemosphere.2020.127279

[75] Kua, T. et al. Aquatic plant, Ipomoea aquatica, as a potential low-cost adsorbent for the effective removal of toxic Methyl Violet 2B dye. *Appl. Water Sci.* **10**, 1–13 (2020).

[76] Lima, E. C., Hosseini-Bandegharaei, A., Moreno-Piraján, J. C. & Anastopoulos, I. A. Critical review of the Estimation of the thermodynamic parameters on adsorption equilibria. Wrong use of equilibrium constant in the van’t hoof equation for calculation of thermodynamic parameters of adsorption. *J. Mol. Liq.* **273**, 425–434. 10.1016/j.molliq.2018.10.048 (2019).

[77] Aguedal, H. et al. Effect of thermal regeneration of diatomite adsorbent on its efficacy for removal of dye from water. *Int. J. Environ. Sci. Technol.* **16**, 113–124 (2019).

[78] Miyah, Y. et al. Assessment of adsorption kinetics for removal potential of crystal Violet dye from aqueous solutions using Moroccan pyrophyllite. *J. Association Arab. Universities Basic. Appl. Sci.* **23**, 20–28 (2017).

[79] Astuti, W., Sulistyaningsih, T., Kusumastuti, E., Thomas, G. Y. R. S. & Kusnadi, R. Y. Thermal conversion of pineapple crown leaf waste to magnetized activated carbon for dye removal. *Bioresour. Technol.* **287**, 121426 (2019). 31103938 10.1016/j.biortech.2019.121426

[80] Hoang, B. N., Nguyen, T. T., Bui, Q. P. T., Bach, L. G., Vo, D. V. N., Trinh, C. D.,… Nguyen, T. D. (2020). Enhanced selective adsorption of cation organic dyes on polyvinyl alcohol/agar/maltodextrin water-resistance biomembrane.* J. Appl. Polymer Sci.*,** 137** (30), 48904.

[81] Loulidi, I., Boukhlifi, F., Ouchabi, M., Amar, A., Jabri, M., Kali, A., … & Aziz, F. Adsorption of crystal violet onto an agricultural waste residue:kinetics, isotherm, thermodynamics, and mechanism of adsorption.*Sci. World J.*, (2020). 10.1155/2020/5873521 PMC7211253 32410908

[82] Elsherif, K. et al. Adsorption of crystal violet dye onto olive leaves powder: Equilibrium and kinetic studies., and Adsorption of crystal violet dye onto olive leaves powder: Equilibrium and kinetic studies.* Chem. Int.*,** 7** (2), 79–89. (2021).

[83] Kyi, P. P., Quansah, J. O., Lee, C. G., Moon, J. K. & Park, S. J. The removal of crystal Violet from textile wastewater using palm kernel shell-derived Biochar. *Appl. Sci.* **10** (7), 2251 (2020).

[84] Li, Y., Xiao, H., Chen, M., Song, Z. & Zhao, Y. Absorbents based on maleic anhydride-modified cellulose fibers/diatomite for dye removal. *J. Mater. Sci.* **49**, 6696–6704 (2014).

[85] Nandi, B. K., Goswami, A., Das, A. K., Mondal, B. & Purkait, M. K. Kinetic and equilibrium studies on the adsorption of crystal Violet dye using Kaolin as an adsorbent. *Sep. Sci. Technol.* **43** (6), 1382–1403 (2008).

[86] Samal, K., Raj, N. & Mohanty, K. Saponin extracted waste biomass of sapindusmukorossi for adsorption of Methyl Violet dye in aqueous system. *Surf. Interfaces*. **14**, 166–174 (2019).

[87] Liu, K. et al. Adsorption of Methyl Violet from aqueous solution using β-cyclodextrin immobilised onto mesoporous silica. *Supramol. Chem.* **33** (4), 107–121 (2021).

[88] Enenebeaku, C. E., Ukaga, I. C., Okorocha, J. N. & Onyeachu, B. I. Adsorption, equilibrium and kinetic studies of the removal of Methyl Violet from aqueous solution using white potato Peel powder. *Int. Lett. Chem. Phys. Astronomy*. **80**, 17–29 (2018).

[89] Foroutan, R., Peighambardoust, S. J., Aghdasinia, H., Mohammadi, R. & Ramavandi, B. Modification of bio-hydroxyapatite generated from waste poultry bone with MgO for purifying Methyl violet-laden liquids. *Environ. Sci. Pollut. Res.* **27**, 44218–44229 (2020). 10.1007/s11356-020-10330-0 32761348

[90] Foroutan, R. et al. Influence of Chitosan and magnetic iron nanoparticles on chromium adsorption behavior of natural clay: adaptive neuro-fuzzy inference modeling. *Int. J. Biol. Macromol.* **151**, 355–365 (2020). 32087228 10.1016/j.ijbiomac.2020.02.202

